# Exposure modality influences viral kinetics but not respiratory outcome of COVID-19 in multiple nonhuman primate species

**DOI:** 10.1371/journal.ppat.1010618

**Published:** 2022-07-05

**Authors:** Alyssa C. Fears, Brandon J. Beddingfield, Nicole R. Chirichella, Nadia Slisarenko, Stephanie Z. Killeen, Rachel K. Redmann, Kelly Goff, Skye Spencer, Breanna Picou, Nadia Golden, Cecily C. Midkiff, Duane J. Bush, Luis M. Branco, Matthew L. Boisen, Hongmei Gao, David C. Montefiori, Robert V. Blair, Lara A. Doyle-Meyers, Kasi Russell-Lodrigue, Nicholas J. Maness, Chad J. Roy

**Affiliations:** 1 Tulane National Primate Research Center, Covington, Louisiana, United States of America; 2 Biomedical Science Training Program, Tulane University School of Medicine, New Orleans, Louisiana, United States of America; 3 Zalgen Labs, LLC, Germantown, Maryland, United States of America; 4 Duke University Medical Center, Duke Human Vaccine Institute, Durham, North Carolina, United States of America; 5 Department of Medicine, Tulane University School of Medicine, New Orleans, Louisiana, United States of America; 6 Department of Microbiology and Immunology, Tulane University School of Medicine, New Orleans, Louisiana, United States of America; University of Pittsburgh, UNITED STATES

## Abstract

The novel coronavirus SARS-CoV-2 emerged in late 2019, rapidly reached pandemic status, and has maintained global ubiquity through the emergence of variants of concern. Efforts to develop animal models have mostly fallen short of recapitulating severe disease, diminishing their utility for research focusing on severe disease pathogenesis and life-saving medical countermeasures. We tested whether route of experimental infection substantially changes COVID-19 disease characteristics in two species of nonhuman primates (*Macaca mulatta*; rhesus macaques; RM, *Chlorocebus atheiops*; African green monkeys; AGM). Species-specific cohorts were experimentally infected with SARS-CoV-2 by either direct mucosal (intratracheal + intranasal) instillation or small particle aerosol in route-discrete subcohorts. Both species demonstrated analogous viral loads in all compartments by either exposure route although the magnitude and duration of viral loading was marginally greater in AGMs than RMs. Clinical onset was nearly immediate (+1dpi) in the mucosal exposure cohort whereas clinical signs and cytokine responses in aerosol exposure animals began +7dpi. Pathologies conserved in both species and both exposure modalities include pulmonary myeloid cell influx, development of pleuritis, and extended lack of regenerative capacity in the pulmonary compartment. Demonstration of conserved pulmonary pathology regardless of species and exposure route expands our understanding of how SARS-CoV-2 infection may lead to ARDS and/or functional lung damage and demonstrates the near clinical response of the nonhuman primate model for anti-fibrotic therapeutic evaluation studies.

## Author summary

The advent of COVID-19’s quick emergence as a human disease necessitated near immediate development of animal models that would recapitulate the viral syndrome to facilitate study of spread, health effects, and evaluation of medications that could either treat or prevent disease. In this study, we compared the health effects of viral infection in two species of nonhuman primates, using two distinct routes of infection which were aerosol or instillation into the nose and upper lung. The results showed that although not effecting overall response in either species of animal, the route by which the experimental infection was performed effected the movement of infectious virus in the animal’s body once infected. These results have implications of what is known about the potential route of transmission of this emerging viral disease.

## Introduction

Since the emergence of SARS-CoV-2 in late 2019, this viral respiratory pathogen has caused over 865,000 deaths in the United States and over 5.6 million deaths worldwide [1,2]. SARS-CoV-2 is the causative agent of Coronavirus Disease 2019 (COVID-19) and produces a range of signs and symptoms from mild cough and anosmia to severe pneumonia accompanied by shortness of breath, with a subset of severely affected individuals experiencing acute respiratory distress syndrome (ARDS) and death [3]. Persistent symptoms can include fatigue, dyspnea, anosmia, and headache [4], which when lasting longer than four weeks beyond resolution of viral persistence has been termed post-acute COVID-19 syndrome (PACS), or ‘long COVID’ [5].

The refinement of COVID-19 disease models using various animal species continues to facilitate the development of highly useful test systems for evaluation and pathogenesis studies [6]. Accordingly, multiple nonhuman primate (NHP) species have been experimentally infected with SARS-CoV-2, with most studies focusing on *Chlorocebus aethiops* (African green monkeys, AGMs) or *Macaca mulatta* (Rhesus macaques, RMs). Other examples of species studied include *Macaca nemestrina* (Southern pigtail macaque) [7], *Macaca leonina* (Northern pigtail macaque) [8], *Macaca fascicularis* (Cynomolgus macaque) [9], *Callithrix jacchus* (Common marmoset) and *Papio hamadryas* (Baboon) [10]. Most of these models involve installation of the virus directly to mucosal surfaces [11,12], though some have included the aerosol modality of exposure [13]. Overwhelmingly, the NHP model of SARS-CoV-2 infection results in a mild to moderate disease, with only one study reporting euthanasia criteria being met post challenge [14]. The RM model of disease, utilized for vaccination [15,16], re-challenge [17], and therapeutic [18] studies, results in disease resolving within three weeks post challenge [9], though some evidence of longer term viral replication has also been reported [19]. AGMs have been utilized for many similar respiratory-based viral diseases including SARS-CoV-1[20], parainfluenza virus [21] and Nipah virus [22]. Their use as SARS-CoV-2 infection models has resulted in observed mild respiratory disease like RMs, but with prolonged shedding of viral RNA [11,13].

Severe disease COVID-19 disease in humans can result in ARDS, characterized by the destruction of the alveolar epithelial lining and significant impairment of gas exchange. Upon survival, the pulmonary space requires substantial repair to regain function, commonly resulting in fibroproliferative disease due to dysregulation of these repair mechanisms. Up to 61% of human autopsies after fatal ARDS show signs of pulmonary fibrosis and 25% of survivors show evidence of restrictive lung disease with long lasting morbidity [23–25]. Within the COVID patient cohort, 42% who develop severe pneumonia will progress to ARDS, with fatal cases generally presenting with pulmonary fibrosis [26,27] or pleuritis [28–31] at autopsy. COVID is characterized by a myeloid cell migration into the lung with a striking accumulation of CD16 expressing monocytes and macrophages within the lungs of humans [32]. Similar myeloid cell infiltration into the lungs has previously been reported in NHP models, and was associated with both a more systemic inflammatory response via serum IL-10:IL-6 ratio and worse disease outcome [33].

In this study, we investigated whether NHP species or exposure modality functionally changes disease course and progression. RM and AGM cohorts were infected via direct mucosal installation (intratracheal/intranasal) or small particle aerosol modality to a low passage SARS-CoV-2 archival (WA1/2020) strain. We demonstrate that infection by small particle aerosol results in slower development of clinical signs of disease and delayed cytokine responses to viral infection in both species. The AGMs revealed prolonged viral dynamics in the respiratory compartment and protracted elimination in the gastrointestinal system in contrast to the RM. Myeloid cell kinetics were defined among the entire cohort to investigate whether variables associated with experimental infection or differences in species susceptibility attribute to the severe outcome of SARS-CoV-2 infection. Pathologic changes were mild and did not correlate with cytokine responses nor myeloid cell kinetics. In the resolving stage of infection, after resolution of most viral RNA, regenerative activity was not observed in the lung of either species, even at sights of ongoing inflammation. These findings indicate the NHP model of SARS-CoV-2 infection may have utility in therapeutic development and evaluation of post-acute COVID sequelae.

## Methods

### Ethics statement

The Tulane University Institutional Animal Care and Use Committee approved all procedures used during this study. The Tulane National Primate Research Center (TNPRC) is accredited by the Association for the Assessment and Accreditation of Laboratory Animal Care (AAALAC no. 000594). The U.S. National Institutes of Health (NIH) Office of Laboratory Animal Welfare number for TNPRC is A3071-01. Tulane University Institutional Biosafety Committee approved all procedures for work in, and removal of samples from, Biosafety Level 3 laboratories.

### Animal cohort and procedures

**Fig 1:**
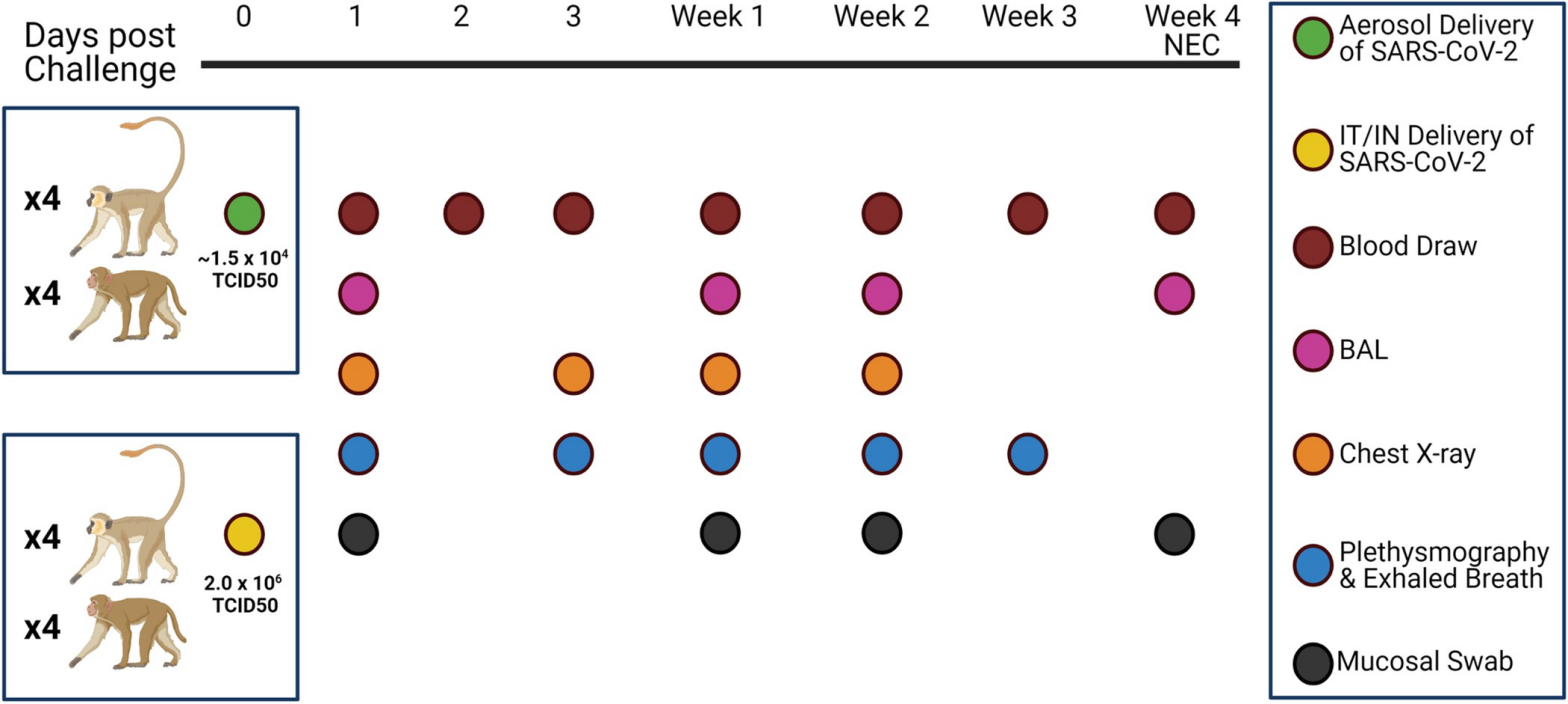
Study Design. SARS-CoV-2 was delivered via aerosol or IT/IN (multiroute) to RMs or AGMs at the noted doses. Animals were followed for 4 weeks, with biosampling performed as indicated above. Fig created in Biorender.com.

A total of 16 NHPs were utilized for this study, between 4 and 11 years old, with most being 7 years of age. All RMs used in this study were captive bred at TNPRC. Four individuals of each species were challenged with SARS-CoV-2 USA_WA1/2020 (World Reference Center for Emerging Viruses and Arboviruses, Galveston, TX), by small particle aerosol, with a mean delivered dose of 1.5 x 10^4^ TCID_50_. The animals exposed to SARS-CoV-2 by aerosol were individually exposed using well-established methodologies as reported in the literature [14,34]. The other four animals of each species were challenged via a combination of intratracheal and intranasal administration (IT/IN), herein referred to as ‘multiroute’, with a delivered dose of 2.0 x 10^6^ TCID_50_ (S1 Table). The total volume delivered during the multiroute experimental infection was 2 ml of viral inoculum. Pre- and post-exposure samples were taken from blood, mucosal (pharyngeal, nasal, rectal) swabs, and BAL supernatant. Chest X-rays were also performed regularly during the study Fig 1. Clinical assessment was performed throughout study. Visual categorical scoring of activity was performed upon daily observation, and respiratory changes during auscultation were categorically scored during physical examination of anesthetized animals at designated timepoints for biosample collection. Animals were monitored for signs of disease throughout the study, with no animals reaching euthanasia criteria. At necropsy 28 days post challenge, mucosal samples were taken, as well as tissues placed in Trizol or z-fix or fresh frozen for later examination.

### Isolation of Viral RNA

RNA was isolated from non-tissue samples using a Zymo Quick RNA Viral Kit (#R1035, Zymo, USA) or Zymo Quick RNA Viral Kit (#D7003, Zymo, USA) for BAL cells, per manufacturer’s instructions. RNA was eluted in RNAse free water. During isolation, the swab was placed into the spin column to elute the entire contents of the swab in each extraction. BAL supernatant was extracted using 100 μL. Viral RNA from tissues was extracted using a RNeasy Mini Kit (#74106, Qiagen, Germany) after homogenization in Trizol and phase separation with chloroform.

### Quantification of viral RNA using quantitative reverse transcriptase PCR

Isolated RNA was analyzed in a QuantStudio 6 (Thermo Scientific, USA) using TaqPath master mix (Thermo Scientific, USA) and appropriate primers/probes (S2 Table) with the following program: 25°C for 2 minutes, 50°C for 15 minutes, 95°C for 2 minutes followed by 40 cycles of 95°C for 3 seconds and 60°C for 30 seconds. Signals were compared to a standard curve generated using *in vitro* transcribed RNA of each sequence diluted from 10^8^ down to 10 copies. Positive controls consisted of SARS-CoV-2 infected VeroE6 cell lysate. Viral copies per swab were calculated by multiplying mean copies per well by amount in the total swab extract, while viral copies in tissue were calculated per μg of RNA extracted from each tissue.

### Live virus quantification

Median Tissue Culture Infectious Dose (TCID_50_) was used to quantify replication-competent virus in swabs and BAL supernatant. VeroE6 ells were plated in 48-well tissue culture treated plates to be subconfluent at time of assay. Cells were washed with serum free DMEM and virus from 50 μL of sample was allowed to adsorb onto the cells for 1 hour at 37°C and 5% CO_2_. After adsorption, cells were overlayed with DMEM containing 2% FBS and 1% Anti/Anti (#15240062, Thermo Scientific, USA). Plates were incubated for 7–10 days before being observed for cytopathic effect (CPE). Any CPE observed relative to control wells was considered positive and used to calculate TCID_50_ by the Reed and Muench method [35].

### Detection of antibodies in serum

The ability of antibodies in serum to disrupt the binding of the receptor binding domain (RBD) of SARS-CoV-2 spike protein to Angiotensin Converting Enzyme (ACE2) was assessed via the Surrogate Virus Neutralization Test (GenScript# L00847) using the included kit protocol modified per the following: Serum samples were diluted from 1:10 to 1:21,870 to determine an IC_50_ for RBD/ACE2 binding. Pseudovirus neutralization testing of matched serum was performed using a spike protein pseudotyped virus in 293/ACE2 cells, with neutralization assessed via reduction in luciferase activity ([36,37]). For binding ELISAs, matched serum was analyzed in duplicate on plates coated with SARS-CoV-2 NP or RBD (Zalgen Diagnostics, Aurora, CO) at 1:100 in diluent. Serum was incubated for 30 minutes at room temperature, washed four times, and incubated with anti-NHP IgG conjugate followed by incubation for 30 minutes at room temp. Development by TMB for ten minutes was followed by stopping of the reaction and reading the plate at 450nm. Positive and negative controls were included in the Surrogate Virus Neutralization Kit. For the pseudovirus assay, negative control included wells with no added serum and positive control of DH1043NHS RBD-specific mAb. For IgG ELISA assays, negative controls were pooled normal plasma, with positive control consisting of pooled immune plasma.

### Serum cytokines

Invitrogen 37-Plex NHP ProcartaPlex kits were purchased and processed according to manufacturer’s instructions with a 1-hour sample incubation period and analysis on the Luminex xMAP. Heatmaps were generated using log_2_-transformed raw fluorescent intensity values input into the R package pheatmap (Raivo Kolde (2019). pheatmap: Pretty Heatmaps. R package version 1.0.12.). Hierarchical clustering was unsupervised.

### BAL cytokines

Invitrogen 37-Plex NHP ProcartaPlex kits were purchased and processed according to manufacturer’s instructions with an overnight sample incubation period and fixation of the plate for one hour in 2% paraformaldehyde before resuspension in Reading Buffer and analysis using the Luminex xMAP. Heatmaps were generated using log_2_-transformed raw fluorescent intensity values input into the R package pheatmap (Raivo Kolde (2019). pheatmap: Pretty Heatmaps. R package version 1.0.12.). Hierarchical clustering was unsupervised.

### Pathology and histopathology

Animals were humanely euthanized following terminal blood collection. The necropsy was performed routinely with collection of organs and tissues of interest in media, fresh frozen, and in fixative. The left and right lungs were imaged and then weighed individually. A postmortem bronchoalveolar lavage (BAL) was performed on the left lower lung lobe. Endoscopic bronchial brushes were used to sample the left and right mainstem bronchi. One section from each of the major left and right lung lobes (anterior, middle, and lower) sample fresh, and the remaining lung tissue was infused with fixative using a 50 mL syringe and saved in fixative. Fixed tissues were processed routinely, embedded in paraffin and cut in 5 μm sections. Sections were stained routinely with hematoxylin and eosin or left unstained for later analysis via fluorescent immunohistochemistry. Trichrome staining was performed as described previously, except with an additional 10 minutes of incubation with Weigert’s Iron Hematoxylin Working Solution [38].

Histopathologic lesions identified in tissues were categorically scored by the same pathologist that performed the necropsies. Lesions were scored based on severity as the lesions being absent (-), minimal (+), mild (++), moderate (+++), or severe (++++). Cohorts were grouped together and non-parametric pairwise comparisons were performed for statistical analysis of histopathologic lesions.

Fluorescent immunohistochemistry was performed on 5 μm sections of Formalin-fixed, paraffin-embedded lung. Sections were incubated for 1 hour with the primary antibodies (SARS, Guinea Pig, (BEI, cat#NR-10361) diluted in NGS at a concentration of 1:1000). Secondary antibodies tagged with Alexa Fluor fluorochromes and diluted 1:1000 in NGS were incubated for 40 minutes. DAPI (4’,6-diamidino-2-phenylindole) was used to label the nuclei of each section. Slides were imaged with Zeiss Axio Scan Z.1 slide scanner. Other antibodies used for fluorescent immunohistochemistry are listed in S3 Table.

### Quantification of fluorescent immunohistochemistry for CD163+ and CD206+

Fluorescent immunohistochemistry was performed on sections of lung. Fluorescently labeled slides were scanned with a digital slide scanner (Axio Scan.Z1, Carl Zeiss Microscopy, White Plains, NY). Phenotypic markers were quantified using digital image analysis software (HighPlex Fl v4.04, HALO, Indica Labs). Cells were first identified by detecting nuclei (DAPI signal) and thresholds for detection of each phenotypic marker were set by our pathologist. The entire lung section was analyzed, and the number of each cellular phenotype (CD163+, CD206+, CD163+CD206+) was reported as cells per mm^2^.

### Flow cytometry

After collection and processing of BAL as previously described, samples were centrifuged, cells resuspended in ammonium chloride potassium (ACK) lysis buffer (#A1049201, Fisher Scientific, USA), and incubated on ice. Media was added to stop lysis and cells were washed before counting and added to 5 mL snap-cap tubes. Cells were stimulated with 0.1% LPS (Enzo Life Sciences Cat# ALX-581-010-L001) and Brefeldin A (BD Bioscience Cat# 555029) overnight (16–18 hours). After stimulation, samples were washed with PBS pH7.2 (Fisher Cat#20012027) and incubated with Fc Block (Tonbo Biosciences Cat# 70-0161-U500) for 5 minutes on ice before viability staining (BD Biosciences Cat# 564406). Cells were washed with Running Buffer (Miltenyi Cat#130-091-221) before incubation with Surface Master Mix for 30 minutes on ice. After subsequent washing with Running Buffer, cells were resuspended in Fixation/Stabilization Buffer (BD Biosciences 554722) for one hour, then washed with Perm/Wash Buffer (BD Bioscience Cat# BDB554723) twice. Intracellular target antibodies were then added and incubated for 20 minutes at room temperature, then washed with Running Buffer. Samples were resuspended in FACS Fixation and Stabilization Buffer (BD Biosciences Cat# 50-620-051) and analyzed within 24 hours or resuspended in PBS and read between 48–72 hours after sample processing. Compensation panels, pooled unstained sample, and unstimulated controls incubated with Brefeldin A only were run with every set of samples.

### Analysis of flow cytometry data

FlowJo version 10.7.1 (BD, Oregon, USA) was used to analyze flow cytometry data. Acquired samples were gated on viability, single cells, CD20/CD3 negativity, and HLA-DR positivity before cell typing. Total alveolar macrophages were gated based on their expression of HLA-DR, CD45, CD163, and CD206. Infiltrating macrophages were gated based on their expression of HLA-DR, CD45, and CD163 along with a lack of CD206 positivity. Monocyte-derived macrophages in the CD163+CD206+ population were distinguished from remaining alveolar macrophages by CCR2 and CD16 positivity. S4 Table lists the antibodies used for staining, and a representative gating strategy S5 Fig).

### TGF-β ELISA

TGF-β1 was quantified in BAL supernatant fluid utilizing a Quantikine ELISA TGF-β1 Immunoassay kit (R&D Systems #BD100B) according to manufacturer’s instructions and utilizing a Sample Activation Kit 1 (R&D Systems #DY010) and Human TGF-β1 Controls (R&D Systems #QC01-1). Each animal’s necropsy BAL supernatant was compared to baseline samples, and log^2^ fold change was calculated.

### Hematology and clinical chemistries

Analysis of blood chemistries was performed using a Sysmex XT-2000i analyzer for EDTA collected plasma, or an Olympus AU400 chemistry analyzer for serum.

## Results

### Viral dynamics

Eight rhesus macaques (RMs) and eight African green monkeys (AGMs) were inoculated with SARS-CoV-2, with a subset (n = 4) of each species experimentally infected either by multiroute (IT/IN) or small particle aerosol. The individuals received 2.0 x 10^6^ TCID_50_ via IT/IN and approximately 1.5 x 10^4^ TCID_50_ via aerosol, ranging from 1.9 x 10^3^ to 7.5 x 10^4^, depending on each animal’s respiratory patterns (S1 Table). Mucosal sampling via pharyngeal, nasal, and rectal swabs, blood, BAL and radiography were performed at indicated times (Fig 1).

**Fig 2:**
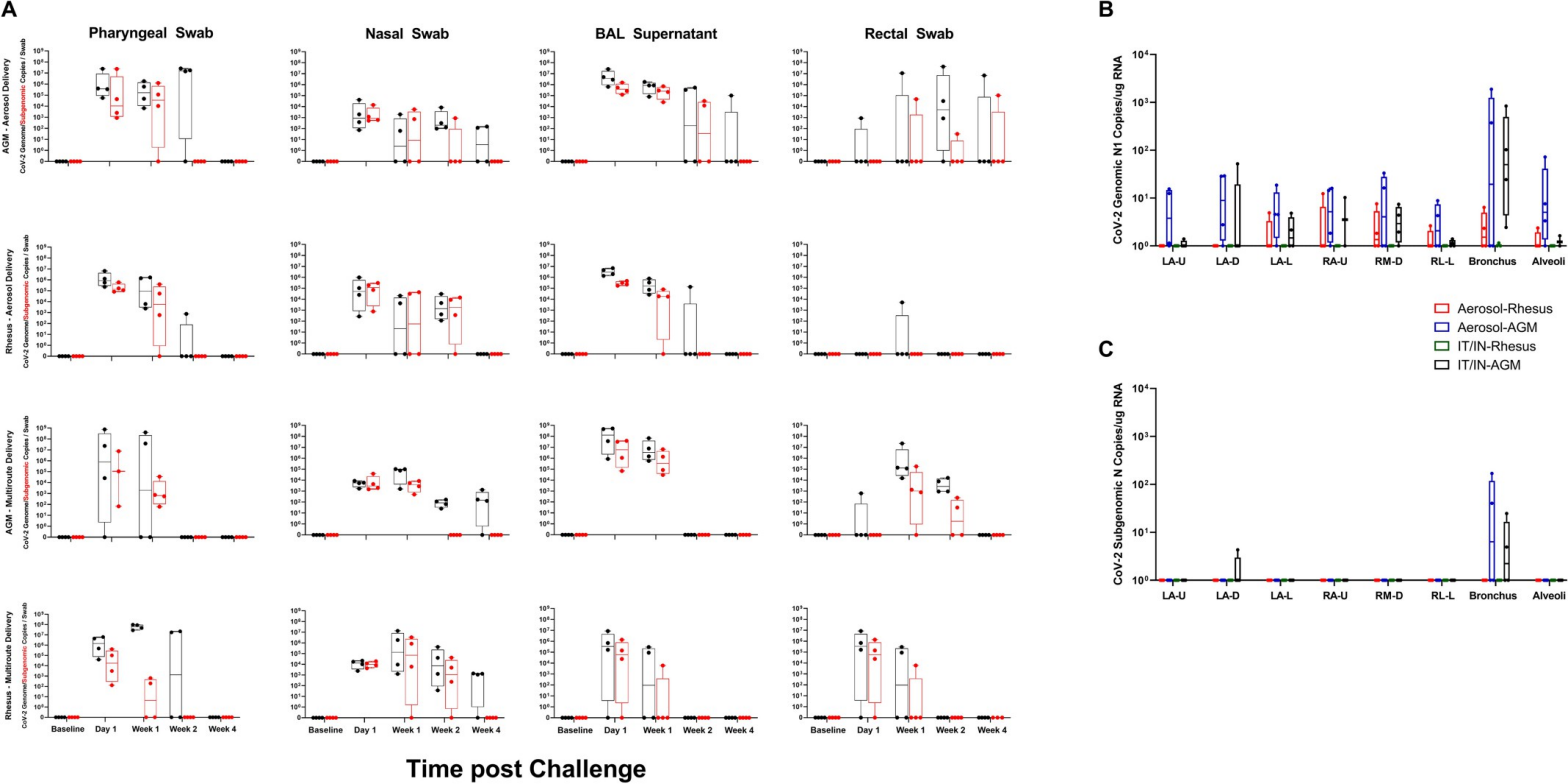
Viral Loads Assessed via RT-qPCR post SARS-CoV-2 Challenge. A)Viral loads in swabs and BAL supernatant were assessed via RT-qPCR post challenge for genomic (black) and subgenomic N (red) RNA. After necropsy, respiratory tissues were analyzed for the presence of genomic (B) and subgenomic (C) content.

Quantitative RT-PCR was used to measure viral load of both genomic and subgenomic vRNA throughout the course of disease (S2 Table). Viral loads were generally at their peak at one day post challenge, with viral RNA trailing off for most species/routes by the end of week 2. All cohorts, except the RM aerosol cohort, had detectable genomic RNA at necropsy in the nasal swabs, though nothing was still detectable in the pharyngeal swab. In the BAL supernatant, genomic RNA persisted longer in the aerosol groups as compared to their species-matched IT/IN exposure cohort, with the AGM aerosol cohort still having detectable genomic vRNA at necropsy, and detectable subgenomic vRNA two weeks post challenge. Persistent, delayed shedding of viral RNA was observed in rectal swabs of the aerosol cohorts across species, with subgenomic vRNA present at necropsy in the AGM aerosol animals (Fig 2A). Overall viral loads, represented as area under the curve of vRNA over the course of the study, overall higher genomic vRNA in the multiroute compared to the aerosol cohort in the RMs, with the same relationship being present in rectal swabs for subgenomic vRNA (S1 Fig).

Genomic RNA was detected in multiple lung regions, with the notable exception of the RM multiroute cohort, where no persistent RNA was found at necropsy (Fig 2B). All AGM animals displayed subgenomic vRNA in lung tissue at necropsy, with detectable amounts of viral RNA in the bronchus and one individual maintaining detectable amounts in the LA-D region (Fig 2C).

Live virus was measured in each sample via the median tissue culture infectious dose (TCID_50_) assay. Viral loads followed a similar pattern of a high peak at one day post challenge, in all animals regardless of exposure modality or species. The aerosol cohort exhibited longer term viral loads, with one AGM still possessing detectable virus at week three post challenge in pharyngeal swab samples. Virus was detected in nasal swabs and BAL supernatant of the AGMs regardless of exposure, with much less detected in the RMs (S2 Fig).

### Antibody responses

**Fig 3:**
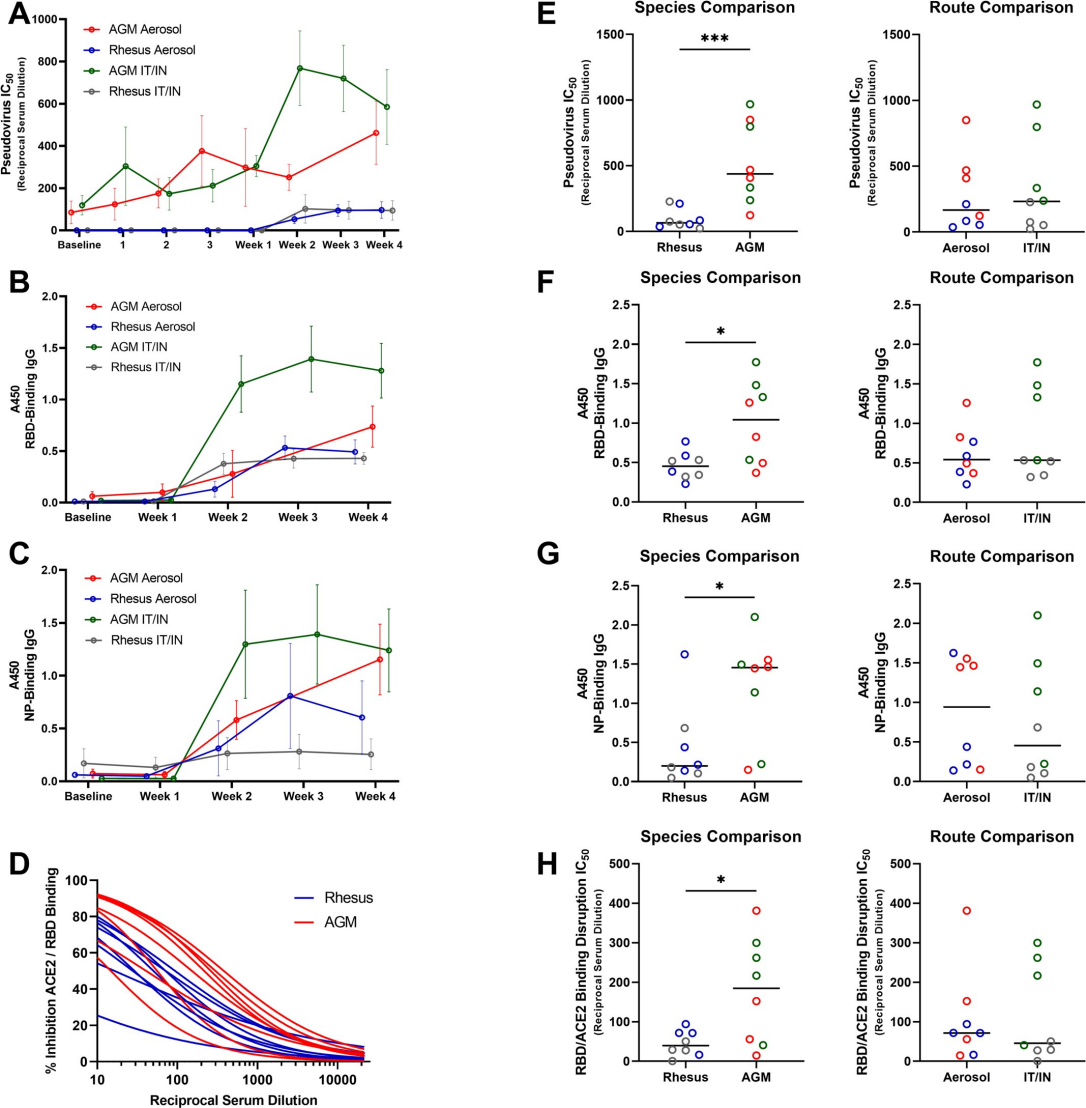
Antibody Responses to SARS-CoV-2 Challenge. Antibody responses were followed post challenge by A)pseudovirus neutralization, B and C) ELISA for binding to RBD and NP, respectively, and D) surrogate virus neutralization test at necropsy. Route and species variability in antibody levels at time of necropsy were compared for each assay type (E-H). Comparisons were made using the Mann-Whitney or Welch’s t-test, depending on normality of data. Asterisks represent significant comparisons (*, p<0.05; ***, p<0.001).

We used a combination of enzyme-linked immunosorbent assay (ELISA) and pseudovirus neutralization to characterize the antibody responses of the cohorts during SARS-CoV-2 infection. In all cases, with pseudovirus neutralization (Fig 3A), and binding of RBD and NP (Fig 3B and 3C, respectively), RMs showed less antibody development than AGMs, regardless of exposure modality, though their anti-RBD development resembled the AGM aerosol cohort (Fig 3B). The RM aerosol cohort displayed anti-NP titers equivalent to the AGM aerosol animals at week three, with a drop in titers at necropsy (Fig 3C). The AGM mutiroute cohort showed the highest magnitude of antibody development, with a peak at week 3, then a slight decrease from peak by necropsy. Notably, the AGM aerosol cohort displayed an overall delayed antibody response, which peaked at necropsy, indicating the potential to continue to increase at later time points. This peak in titers was observed in AGMs in both exposure cohorts (Fig 3D). In all cases, a higher titer was seen at necropsy in the AGM cohort regardless of exposure modality than the RM cohort, with no difference observed based on exposure modality (Fig 3E–3H).

### BAL and serum cytokines

**Fig 4:**
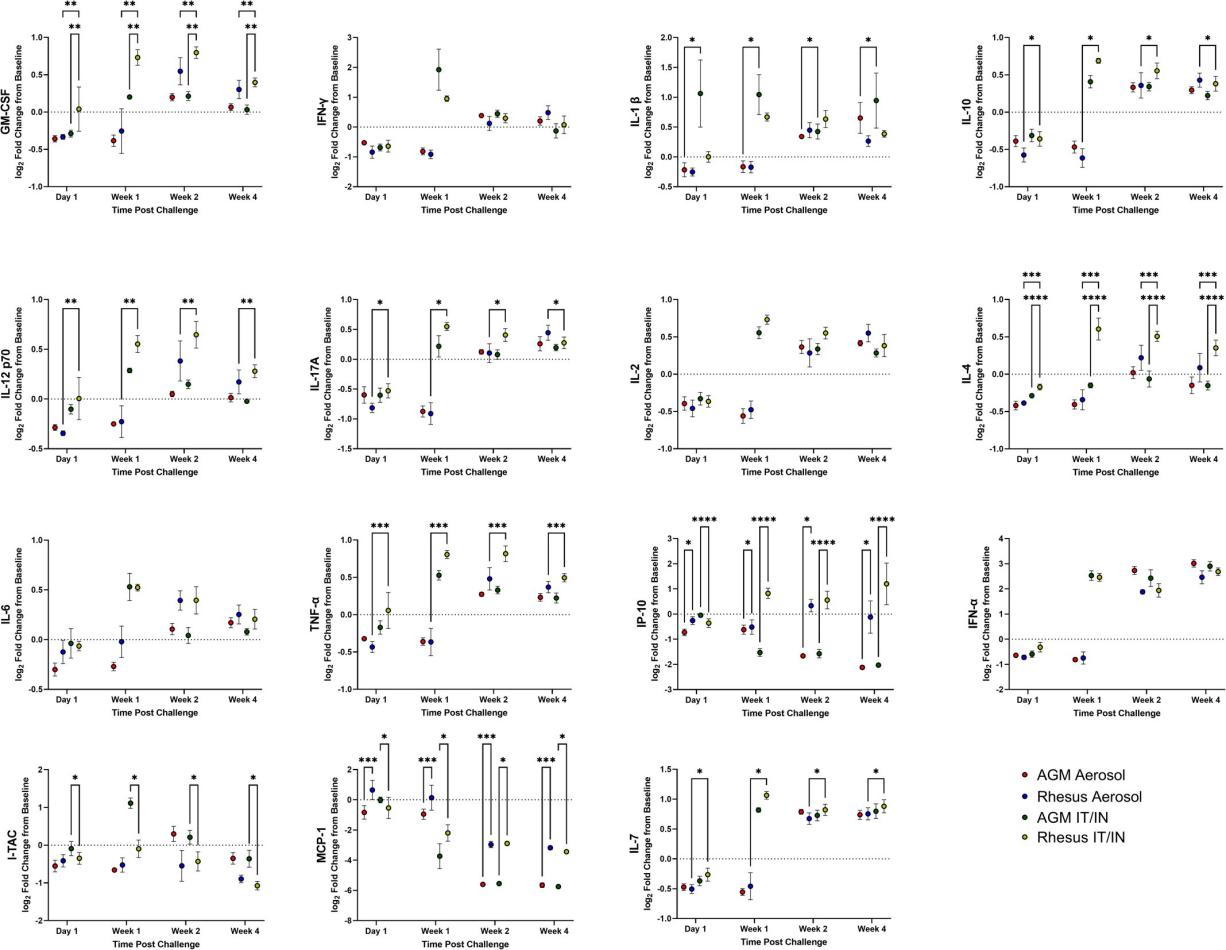
BAL Supernatant Cytokines post SARS-CoV-2 Challenge. Cytokines in BAL supernatant were analyzed at indicated time points post challenge. Comparisons were made with two-way ANOVA using Tukey’s multiple comparisons test. Asterisks represent significant comparisons (*, p<0.05; **, p<0.01;***, p<0.001).

**Fig 5:**
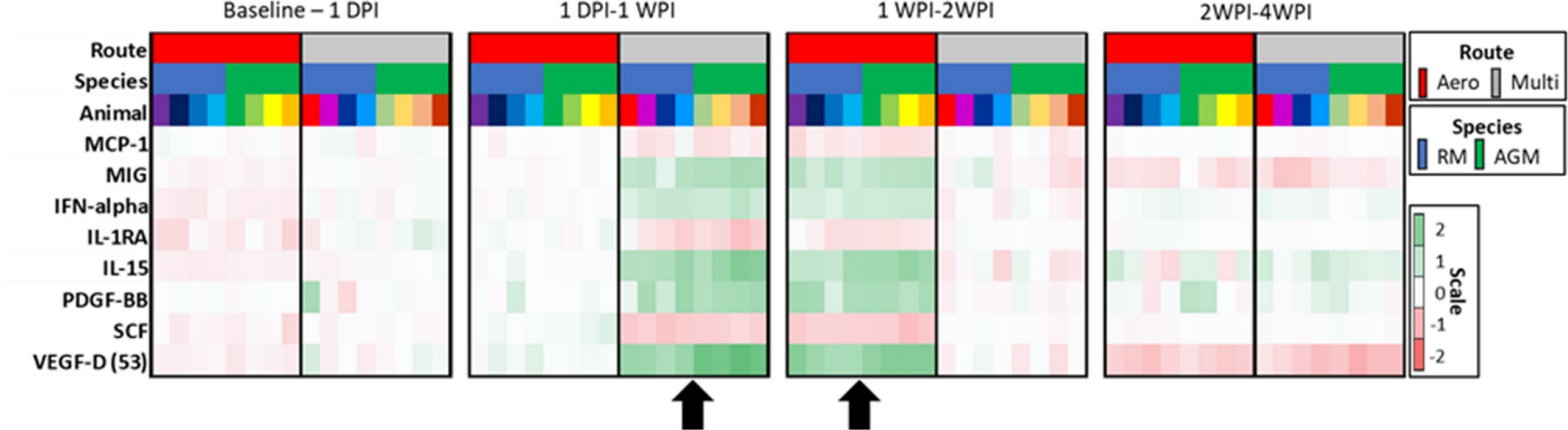
Heat map of BAL cytokine expression dynamics. Changes in the cytokine expression between time points demonstrates the delayed cytokine kinetics in the aerosol cohort (red). Similar dynamics are observed in both cohorts (arrows) occurring between 1 day post infection (DPI) and 1 week post infection (WPI) in the multiroute cohort compared to 1–2 WPI in the aerosol cohort.

Measurements of cytokines in serum and BAL supernatant samples were performed throughout the study. Cytokines in the BAL supernatant was more differentially expressed than those in the serum. Changes in BAL cytokine expression was more pronounced in the mutiroute than the aerosol cohort, with the aerosol cohort revealing delayed kinetics. In some cases, the aerosol cytokines continued to trend at necropsy with increases of IL-6, IFN-α, IL-1β throughout sampling. AGMs displayed higher levels of I-TAC and lower levels of MCP-1 than RMs, though trends were more associated with exposure modality than species in the BAL (Fig 4). Cytokine kinetics correlated with route of exposure regardless of species demonstrated by the dynamic (change in log2FC between time points) coincident increases and decreases of several cytokines. This included dynamic changes in expression of MCP-1, MIG, IFN-a, IL-1RA, IL15, PDGF-BB, SCF, and VEGF-D with lesser but still coincident changes in sCD40L, FGF-2, BDNF, SDF-1. The coincident decreases in expression of IL-1RA and MCP-1 combined with increases in IFN-a, IL-15, PDGF-BB, VEGF-D, MIG indicate an enhanced antiviral response with a switch from proinflammatory to resolution responses at their respective time points in each cohort. In the multiroute cohort these changes occurred between 1dpi and 1 wpi, compared to the aerosol cohort where near identical changes occurred between 1wpi and 2 wpi (Fig 5). The RM multiroute cohort exhibited a larger increase in some cytokines than the AGM cohort, including GM-CSF, IL-10, IL-4, and IP-10, and an increase above the RM aerosol animals in IL-12p70, IL-17A, TNF-α and IL-7. The aerosol cohort exhibited lower levels of MCP-1, with the aerosol-exposed RMs expressing IP-10 more highly than the AGM aerosol animals.

Serum cytokines were measured throughout the study as well. Here, the RM multiroute animals displayed lower levels of GM-CSF, IL-5, IL-6, and MCP-1. The temporal differences in exposure modality were not present in the serum samples. The AGM mutiroute displayed higher levels of MIG, MIP-1β and VEGF-A (S3 Fig).

### Clinical scoring

**Fig 6:**
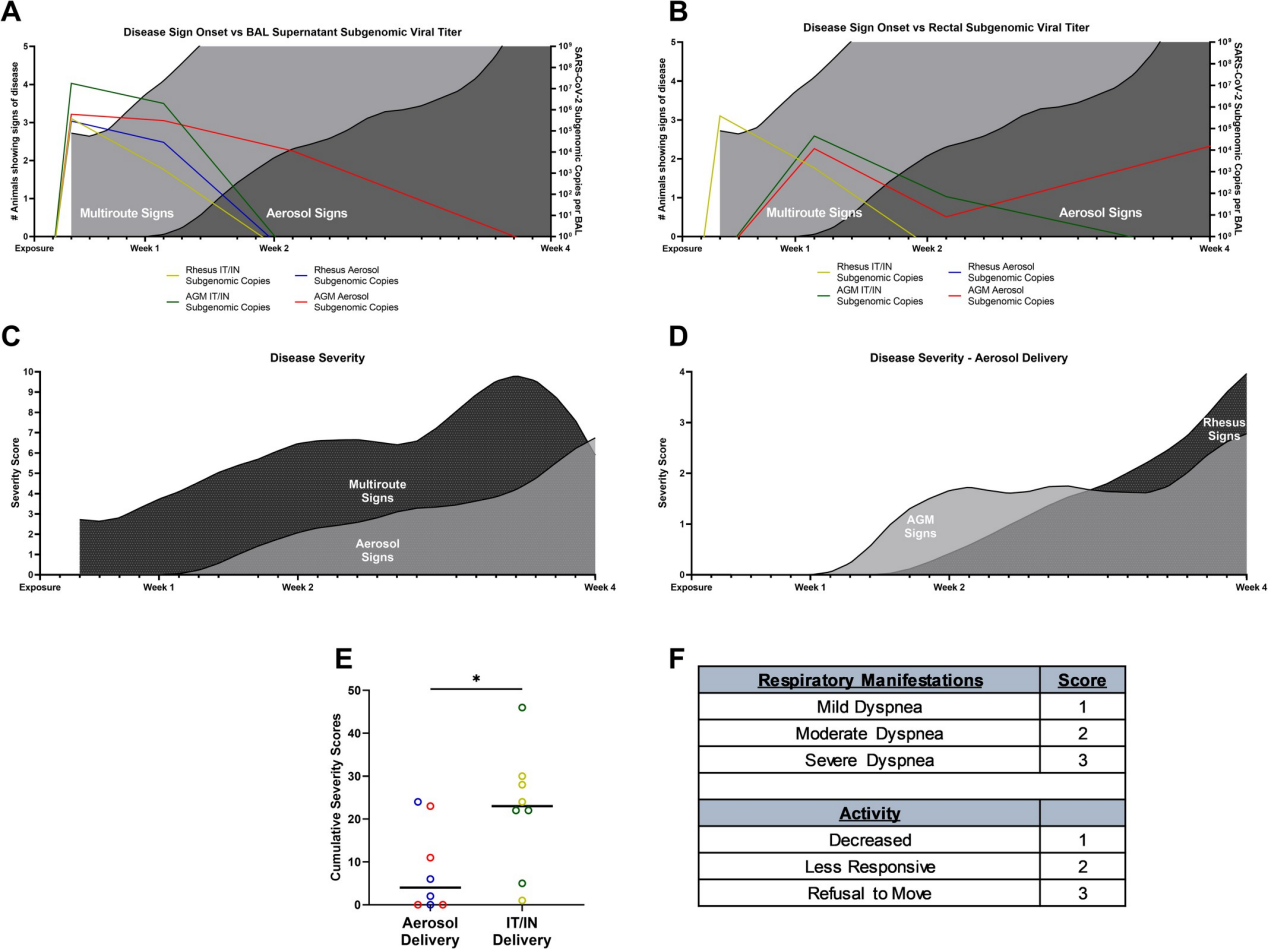
Clinical Disease Severity Scores. Signs of disease present in each cohort over time. Number of animals showing signs of disease along with subgenomic viral loads in A) BAL supernatant and B) rectal swabs. Severity scores per group in C) both delivery cohorts and D) aerosol cohort split into species. E) Cumulative severity scores per delivery cohort. F) Simplified scoring system used for cohorts. Curves in figures were smoothed. Comparisons made using Welch’s t-test. Asterisks represent significant comparisons (*, p<0.05).

Clinical observations were performed throughout the study and resulted in categorical scores as a corollary to clinical disease development in the NHPs. The number of animals showing signs of SARS-CoV-2 related disease increased rapidly early during the study in the mutiroute cohort in either species, continued to a peak at week three post challenge, and then declined by day 28 termination of the experiment (necropsy). This contrasts with aerosol-exposed animals, which did not display signs of disease until one week post challenge, and thereafter continued to increase until day 28 at necropsy (Fig 6A and 6B). The same pattern was observed in overall group severity scores (Fig 6C). Overlaying the clinical score curves with PCR data showed persistent subgenomic vRNA in the BAL supernatant (Fig 6A) and rectal swabs (Fig 6B) of the AGM aerosol cohort, suggesting a slower onset, more persistent disease process. Both species exhibit persistent signs of disease, with RMs peaking than AGMs at necropsy over that of the AGM animals (Fig 6D) when data is segregated by species among all aerosol-exposed animals. Overall, the mutiroute cohort exhibited a higher cumulative score than the aerosol cohort, though the latter cohort’s clinical signs continued to increase until the termination of the study (Fig 6E). The scoring system was based on respiratory signs and changes in activity (Fig 6F).

### Pleuritis

**Fig 7:**
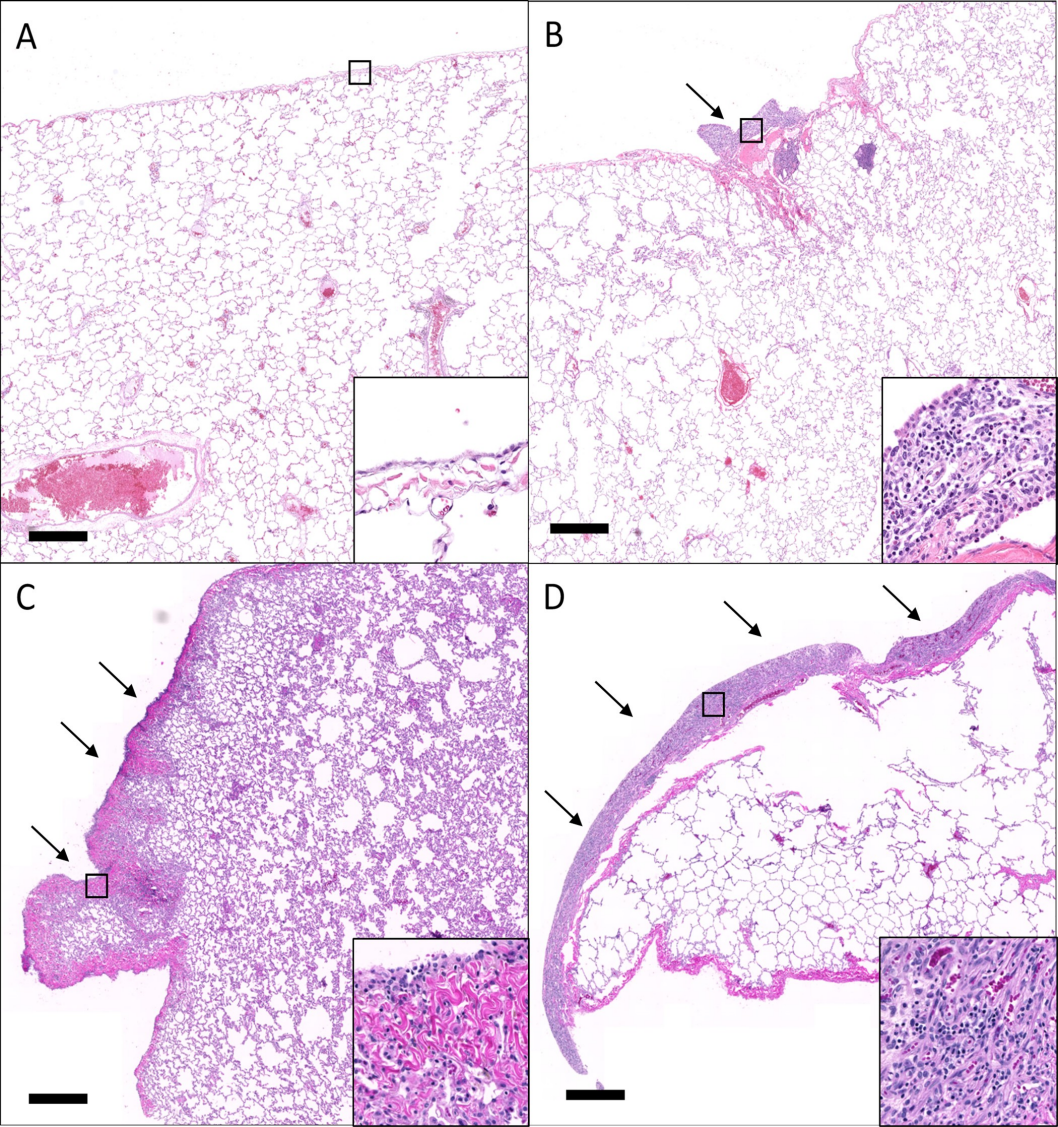
Pleuritis in SARS-CoV-2 infected animals. A) Normal pleura from a naïve animal. B-D). SARS-CoV-2 infected animals exhibited variable pleuritis (arrow) ranging from minimal (B) to mild (C) to moderate (D). Insets of the boxed regions show the magnitude of thickening and infiltration by inflammatory cells. H&E. Bar = 1 mm.

Seven animals of the sixteen exposed to SARS-CoV-2 infection by either aerosol or mutiroute developed pleuritis of varying but generally mild degree, including animals with no signs of clinical disease observed (Fig 7A–7D). Pleuritis was often localized to one or two sections in an animal, and was equally distributed throughout the lung (anterior, middle, and lower; right and left lung lobes). These localized areas of pleuritis may represent previously resolved sites of SARS-CoV-2 infection which is known to have a patchy distribution and is often found in subpleural regions [39]. Immunohistochemistry for SARS-CoV-2 failed to identify viral proteins at the time of necropsy. This is consistent with previous reports that showed detection of virus after the acute phase of infection can be challenging [40].

### Pulmonary influx of macrophages in SARS-CoV-2 infection

**Fig 8:**
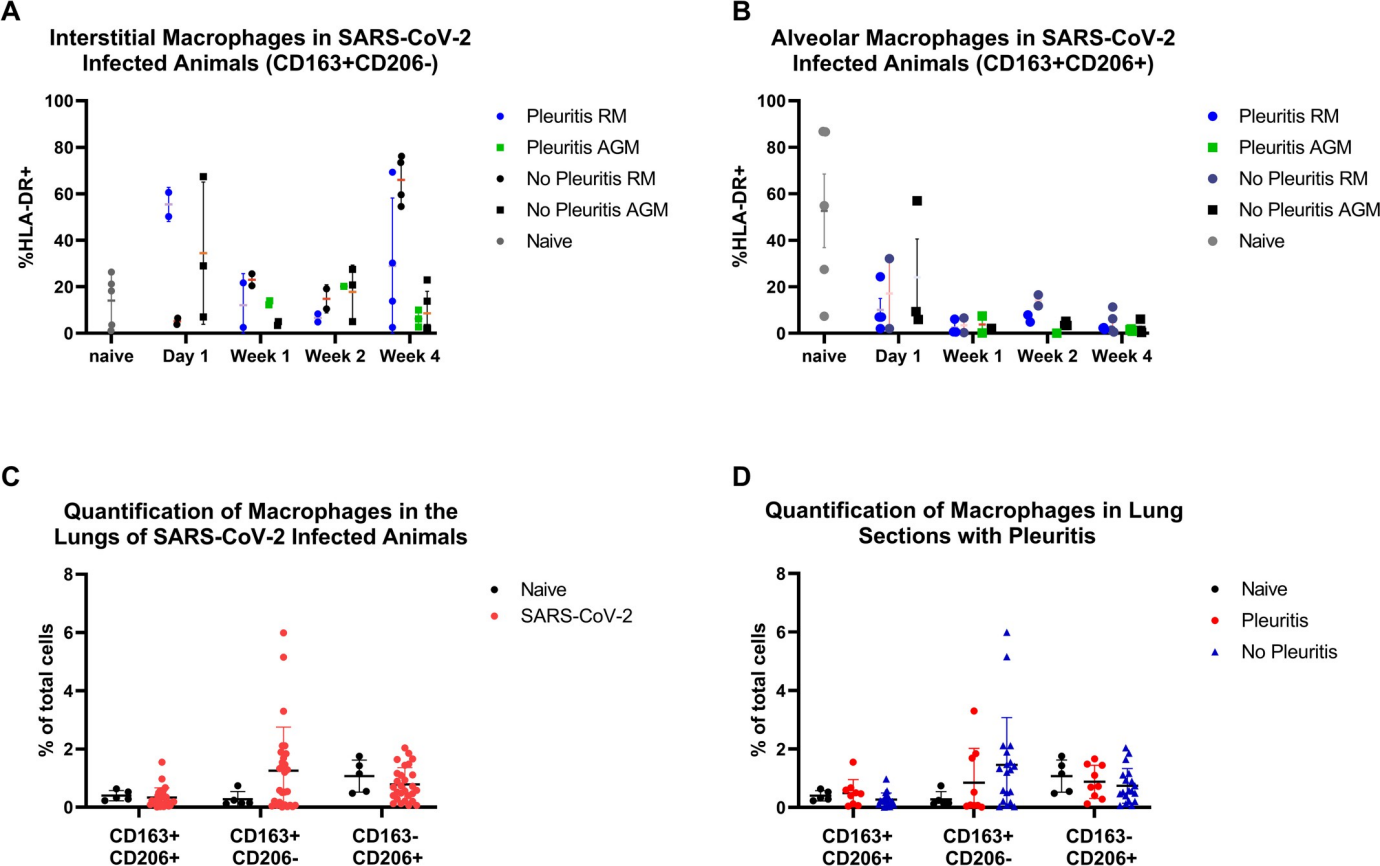
Myeloid cell kinetics. A) Flow cytometry of BAL fluid showed a decrease in alveolar macrophages in SARS-CoV-2 infected animals over the course of the study. The decrease in alveolar macrophages was more pronounced in animals with pleuritic (red). B) Infiltrating interstitial macrophages (CD163+CD206-) were elevated compared to naïve animals both early (d1) and late (necropsy). C).

Flow cytometric analysis of CD163+CD206+ alveolar macrophages in BAL fluid revealed an average decrease in alveolar macrophages at day one post-exposure that continued to decline throughout the study (Fig 8B). Interstitial macrophages classified as CD163+CD206- were higher at day one than in naïve comparators, and resolved by week one with a few animals showing elevation at necropsy (Fig 8A). There was no significant difference in macrophage subpopulation kinetics between animals that developed pleuritis and those that did not. Additionally, quantitative analysis (HiPlex FL v4.04, HALO, Indica Labs) of fluorescent immunohistochemistry (FIHC) utilizing the same markers (CD163+ and CD206+) was performed to compare with flow cytometry findings. SARS-CoV-2 infected animals showed increased CD163+CD206- macrophages in lung sections by FIHC compared to naïve controls (Fig 8C), although this difference did not achieve statistical significance (p = 0.15, T-test). The FIHC data supports the flow cytometry data which showed a similar elevation in CD163+CD206- subpopulation at necropsy, without a difference between animals that developed pleuritis (Fig 8D). The decrease in alveolar macrophages (CD163+CD206+ cells) observed with flow cytometry was not observed in the FIHC analysis, although this may be due to how the numbers were reported: FIHC (proportion of all cells within the lung) compared to flow cytometry (proportion of HLA-DR+ cells within BAL fluid).

### Long-term myofibroblast persistence and lack of regenerative activity in areas of pleuritis

**Fig 9:**
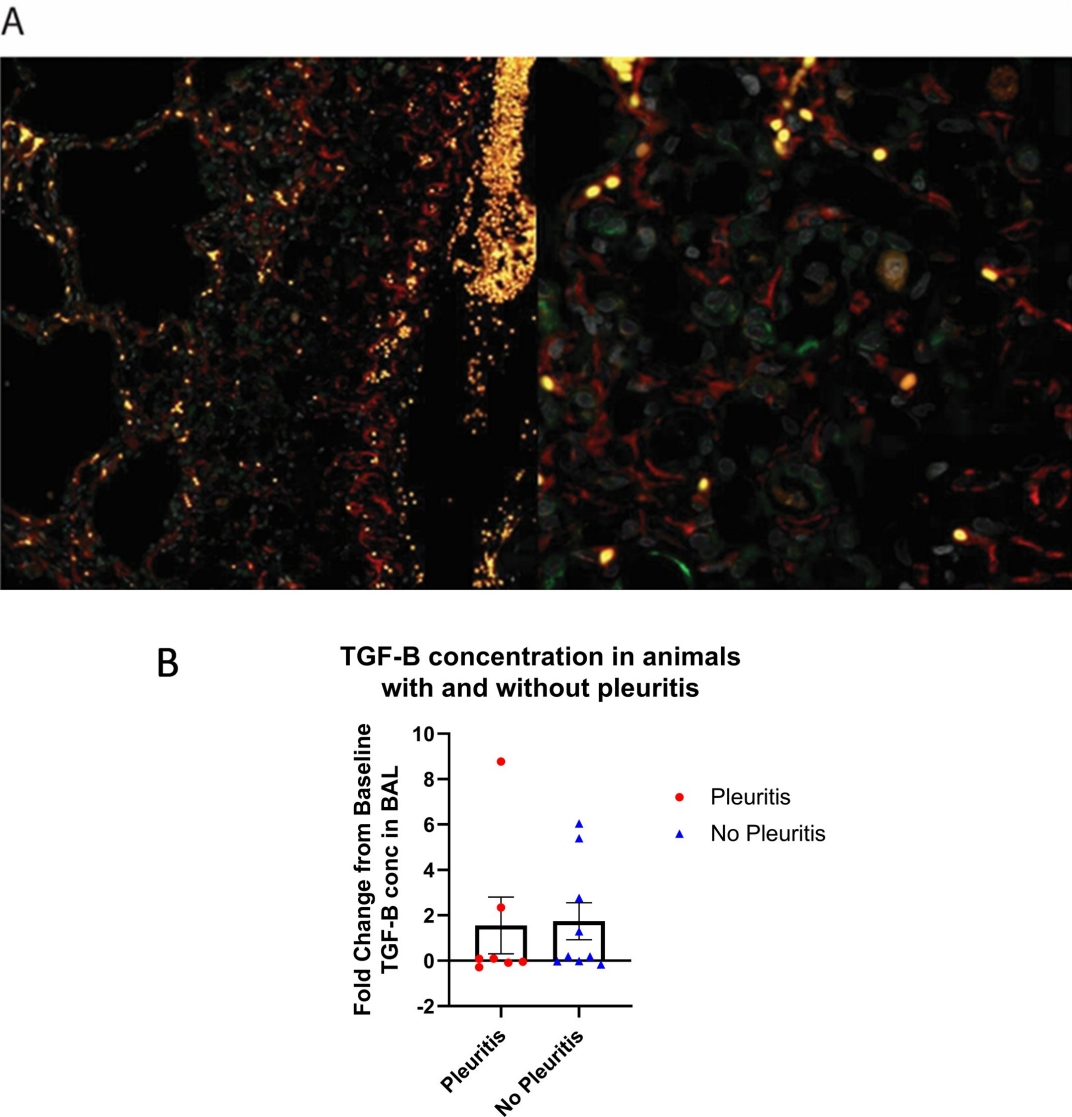
Activated myofibroblasts are present within scarred lung tissue and persist long term. A) KN90, lung alveoli. Myofibroblasts characterized by double-positive staining of αSMA (red) and cytokeratin 5 (green). Bar = 100μm. B) There was a >2 fold increase above baseline in TGF-B concentration in BAL in five of sixteen animals. TGF-B concentration was not siginifcantly different between animals that developed pleuritis.

**Fig 10:**
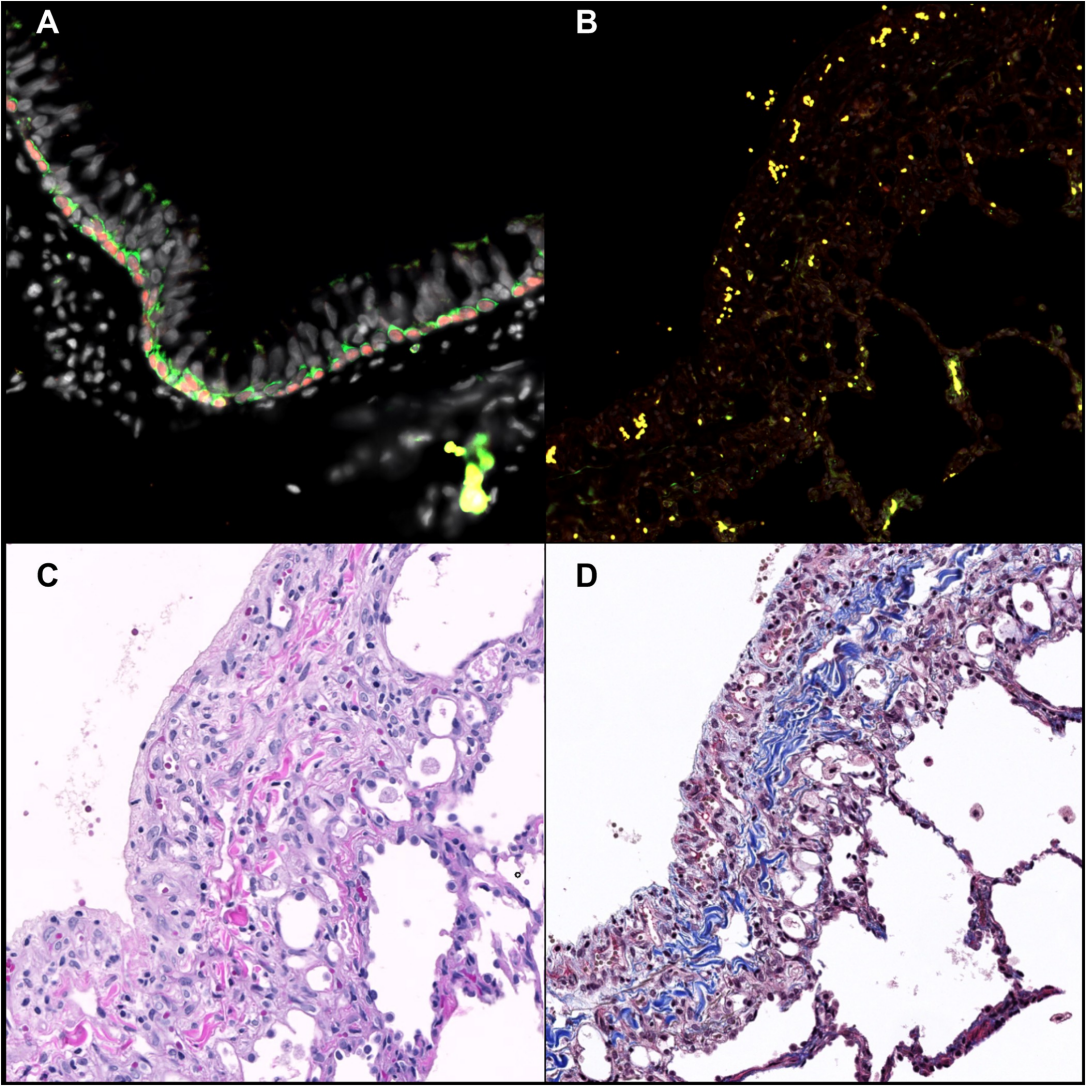
Absence of regenerative activity at 28 days post-exposure. Immunofluorescence for the detection of bronchial epithelial progenitor cells. A) NJ48, lung. The epithelial lining of large airways (bronchi) has a basal layer containing progenitor cells characterized by cytoplasmic expression of cytokeratin 5 (green) and nuclear expression of p63 (red). B-D) KN90, lung alveoli. B) Progenitor cells are not observed in regions of pleuritis and pleural fibrosis with H&E (C) and trichrome (D). Bar = 100μm.

Pleuritis is the result of inflammatory cell infiltration along the margin of the lung and is often associated with fibrosis and thickening of the pleura. To determine if activated myofibroblasts were present within regions of pleuritis, lung sections of animals both with and without pleuritis were stained with fluorescent markers for αSMA and cytokeratin 5. Myofibroblasts were present in areas of collagen deposition (Fig 9A). Myofibroblasts are activated by TGF-β, which is produced by macrophages and allows for their continued production and deposition of collagen. Additionally, TGF-β signaling has also been implicated in fibrogenesis [41,42]. To determine if TGF-β in BAL fluid could be used as a biomarker for pleuritis we used an ELISA assay to measure the levels of TGF-β in BAL supernatant at baseline and at necropsy. There was no significant difference in TGF-β concentration in BAL between animals with and without pleuritis (Fig 9B). Although some animals did show elevated concentrations, the presence of TGF-β was not discernable between outcome, species, nor modality of SARS-CoV-2 infection. Because TGF-β is closely involved in wound healing and associated immunoregulation at sites of injury, there was interest in understanding whether regenerative processes were taking place in animals recovering from acute viral infection. Fluorescent antibodies p63 and cytokeratin 5 were used to identify basal lung progenitor cells (p63+CK5+) to measure whether regenerative potential within the lung was underway. We failed to identify a regenerative response by measure of basal lung progenitor cell activity after SARS-CoV-2 infection, even at sites with pleuritis (Fig 10A–10D). This finding either suggests that this regenerative pathway is not engaged in response to SARS-CoV-2 infection or that infection may hamper the regenerative response in the lung of experimentally infected macaques.

### Hematology and clinical chemistries

Complete blood counts and clinical chemistries were performed throughout the study at times of blood collection. The AGM mutiroute cohort displayed lower levels of neutrophils than the AGM aerosol animals during the study (S6C Fig) and higher levels of monocytes than the AGM aerosol and RM multiroute cohorts (S6F Fig). Blood urea nitrogen was elevated in the AGM aerosol cohort compared to the RM aerosol cohort (S7D Fig), though overall increases in BUN were only slightly higher than expected levels for either NHP species.

## Discussion

SARS-CoV-2 infection continues to result in morbidity and mortality in large segments of the population, with some long-term sequelae persisting well beyond viral clearance [1,43–47]. Variants of concern (VOC) are arising that threaten to continue the pandemic into the future to an uncertain degree, necessitating efforts that transcend vaccination strategies alone, and promote alternative prophylactic and therapeutic development both for acute infection and persistent symptoms [48–52]. Multifocal medical countermeasure development concurrent with viral evolution necessitates a suitable animal infection model that recapitulates the immunologic and clinically relevant aspects of COVID-19. There have been a number of accounts of exploratory COVID-19 model development in various nonhuman primate species [6,11,13,17,53–55], however this work is the only that directly compares two prominent species while comparing two distinct exposure routes employed for experimental infection. Accordingly, we evaluated two distinct exposure routes: concomitant intratracheal and intranasal (multiroute), and small particle aerosol in the rhesus macaque (RM) and African green monkey (AGM). The ‘mutiroute’ group viral dose was based upon volume applied in the combined intratracheal and intranasal administration. Reaching the equivalent dose (2E+06 TCID_50_) by the aerosol route was hampered by relatively low-titer virus (3E+06 TCID_50_) and the logistics of producing aerosols for the purposes of individual exposure. The natural dilution and corresponding efficiency of aerosolization of SARS-CoV-2 affects the resultant dose to each animal, with a net effect of a nearly 2-log disparity between the multiroute and aerosol groups. This disparity should be considered when any direct comparisons are made considering only route of exposure.

Animals in the mutiroute exposure group displayed earlier and increased respiratory signs of disease, as well as a higher cumulative categorical clinical score than the aerosol cohort. The aerosol exposure group, in contrast, began showing signs of disease a full week later. Though signs of disease were delayed in the aerosol cohort, viral titers were similar beginning day one through week one post-exposure in all in all groups, with persistent infection detected in aerosol exposure cohorts through end of the study. The similarity of the pattern of early viral kinetics between exposure groups despite differences in clinical signs of disease suggests that viral replication may not be the direct cause of onset of signs of disease, but rather the immune response to infection may play a larger role, as exemplified by delayed BAL cytokine kinetics as observed in the aerosol cohort. Additionally, animals who did not display signs of disease throughout the study nonetheless exhibited detectable measures of viral replication, including PCR detection of viral genomic and subgenomic RNA, indicating that these animals may still be able to transmit virus. Higher viral titers in the lower respiratory tract were observed in AGMs, whereas nasal cavity titer was greater in RMs, representing potential differences in natural aerosol transmission between species.

Systemic inflammatory cytokine/chemokine response including TNFα, IL-6, MCP-1, IP-10, and MIP-1α has been associated with more severe disease in human studies of COVID-19 [3,56]. We observed mild increases in some of these mediators early in infection in serum including MCP-1 and MIP-1α in AGMs of both exposure routes, indicating mobilization and recruitment of monocytes, dendritic cells, and NK cells in these animals. However, systemic inflammatory mediators were not significantly elevated in any of the animals, consistent with mild disease outcomes and lack of the severe disease manifestations reported in human studies of COVID-19.

Localized soluble mediator responses varied among groups and species. More pronounced changes were observed in the BAL than in serum, indicating a more localized pulmonary than systemic response to infection in this model. The mutiroute group in both species showed more pronounced changes than the aerosol cohort, possibly due to dose differences. Similar increases in IL-2, IL-4, IL-6, IL-7, IL-10, TNFα, and IFNγ were observed at week one in mutiroute exposure groups, though the aerosol group saw similar increases a week later. This shift in cytokine response correlates with the shift observed with signs of disease, indicating a delayed immune response to infection despite similar viral kinetics.

Myofibroblast activity has been described within SARS-CoV-2-infected lungs [57] and activated myofibroblasts (αSMA+CK5+) were identified in this study at sites of pleuritis. Myofibroblasts often co-localized with macrophages, further suggesting the role of macrophages in the activation and persistence of myofibroblasts after SARS-CoV-2 infection. TGF-β, a cytokine which induces activation of myofibroblasts and is associated with wound healing, has previously been shown to be elevated in human cases of severe COVID [58]. When the animals reported herein were assayed for TFG-β in BAL fluid at necropsy, a >2-fold increase above baseline was observed in several SARS-CoV-2 infected animals; however, this did not correlate with pleuritis. This lack of correlation may be due to the comparatively mild pulmonary disease observed in this study as compared to human cases of severe COVID. Further studies should explore the secretion and resolution of TGF-β production and its relation to pulmonary pathology after SARS-CoV-2 infection. Immunohistochemistry to identify basal progenitor cells (p63+CK5+) showed minimal difference in the frequency of progenitor cells between animals naïve to SARS-CoV-2 and infected animals with or without pulmonary pathology. This lack of regenerative activity raises the concern that SARS-CoV-2 may negatively affect the regenerative capacity of the lung or lead to persistent pulmonary pathology. These findings suggest the NHP model of SARS-CoV-2 infection may provide a useful tool for studying post-acute sequalae.

Delayed local pulmonary cytokine/chemokine responses and prolonged viral titers through necropsy may indicate that aerosol inoculation produces prolonged consequences of infection. Further, broad dose differences (nearly 2-log) between mutiroute and aerosol groups produce similar outcomes, which suggests aerosol exposure is a more potent exposure modality for SARS-CoV-2 infection. The aerosol AGM group also showed consistent, modest signs of disease trending upward at necropsy, persistent viral titers in the BAL, and a trend of increasing inflammatory cytokines at necropsy, indicating aerosol exposure of AGMs as an appropriate NHP model of post-COVID syndrome.

Both aerosol RM and mutiroute AGM groups demonstrate increased respiratory rate, comparable neutrophilia, and increased signs of disease in their respective exposure group. However, the AGM mutiroute group exhibited the greatest lymphocyte and monocyte increases, earliest and greatest signs of disease, highest pharyngeal peak viral titer, most inflammatory cytokines in serum, and most consistently high BAL inflammatory cytokines (IL-6, IFNα, TNFα, IL-1β). Taken together, these responses suggest mutiroute exposure of AGMs most accurately recapitulates human disease with poorer clinical outcome. Future studies should consider these findings when determining species and exposure modality for the exploration of currently identified or future VOC to combat the COVID-19 pandemic.

## Supporting information

S1 Fig Viral Loads Assessed via RT-qPCR post SARS-CoV-2 Challenge. Viral loads, assessed by RT-qPCR for genomic and subgenomic RNA, represented as area under the curve for the post challenge period. Comparisons between groups were made via Kruskal-Wallis with Dunn’s multiple comparisons test. Asterisks represent significant comparisons (*, p<0.05).
(TIF)

S2 Fig Viral Loads Assessed via TCID50 post SARS-CoV-2 Challenge. Viral loads, assessed by TCID_50_, represented as area under the curve for the post challenge period. Black lines indicate viral loads per individual, with red lines indicating group geometric means. Dotted lines indicate a positive sample below the limit of quantification.
(TIF)

S3 Fig Serum Cytokines post SARS-CoV-2 Challenge. Cytokines circulating in serum were analyzed at indicated time points post challenge, with early indicating a mean value of days 1, 2 and 3 post challenge. Comparisons were made with two-way ANOVA using Tukey’s multiple comparisons test. Asterisks represent significant comparisons (*, p<0.05; **, p<0.01;****, p<0.0001).
(TIF)

S4 Fig Representative histopathology. A,B: Aerosol RM, right middle lobe. A) The pleura is segmentally thickened (pleuritis, arrows). B) Regions of pleuritis are characterized by fibrosis (dotted line) with infiltration by mononuclear cells. Aggregates of similar inflammatory cells are present subpleurally (arrow). C,D: Aerosol AGM, left anterior lobe. C) The pleura is segmentally thickened (arrows). D) The pleura is lined by hypertrophic mesothelial cells (arrow) and there is infiltration of the subpleural parenchyma by histiocytes. E,F: IT/IN RM, left lower lobe. E) The pleura is segmentally thickened (pleuritis, arrows). F) The pleura is thickened by fibrosis and infiltrated by mononuclear cells, predominately lymphocytes. G,H: IT/IN AGM, right lower lobe. G) There is mild congestion and rare perivascular inflammation (arrow). H) Perivascular inflammation is characterized by infiltration of the tunica adventitia by mononuclear cells.
(TIF)

S5 Fig BAL flow cytometry gating strategy. Representative gating strategy to classify alveolar (CD163+CD206+), interstitial (CD163+CD206-), monocyte-derived (CD163+CD206+CD16+CCR2+), and resident alveolar (CD163+CD206+CD16-) macrophages in BAL.
(TIF)

S6 Fig Hematology-Based Parameters of SARS-CoV-2 Challenge. Complete blood counts were performed at indicated times and were compared for counts of RBCs, platelets, neutrophils, lymphocytes, WBCs and monocytes (A, B, C, D, E, and F, respectively), as well as neutrophil/lymphocytes ratio (G). Comparisons were made via two-way ANOVA with Tukey’s multiple comparisons test. Asterisks represent significant comparisons (****, p<0.0001).
(TIF)

S7 Fig Clinical Chemistry-Based Parameters of SARS-CoV-2 Challenge. Clinical chemistries were performed at the indicated times post challenge. Comparisons between each group were made for log_2_ fold change from baseline of creatinine (A), ALT (B), BUN (D) and concentrations of CRP (C) and AST (E). Comparisons were made via two-way ANOVA with Tukey’s multiple comparisons test. Asterisks represent significant comparisons (**, p<0.01).
(TIF)

S1 Table Study Animal Characteristics. (TIF)

S2 Table RT-qPCR Primers and Probes. (TIF)

S3 Table Antibodies used for fluorescent immunochemistry. (TIF)

S4 Table Antibodies used for flow cytometry analysis. (TIF)
