# Supplementary figures and images for: Exposure modality influences viral kinetics but not respiratory outcome of COVID-19 in multiple nonhuman primate species
Source: PLoS Pathog. 2022 Jul 5;18(7):e1010618. doi: 10.1371/journal.ppat.1010618 (PMC9286241; doi:10.1371/journal.ppat.1010618)

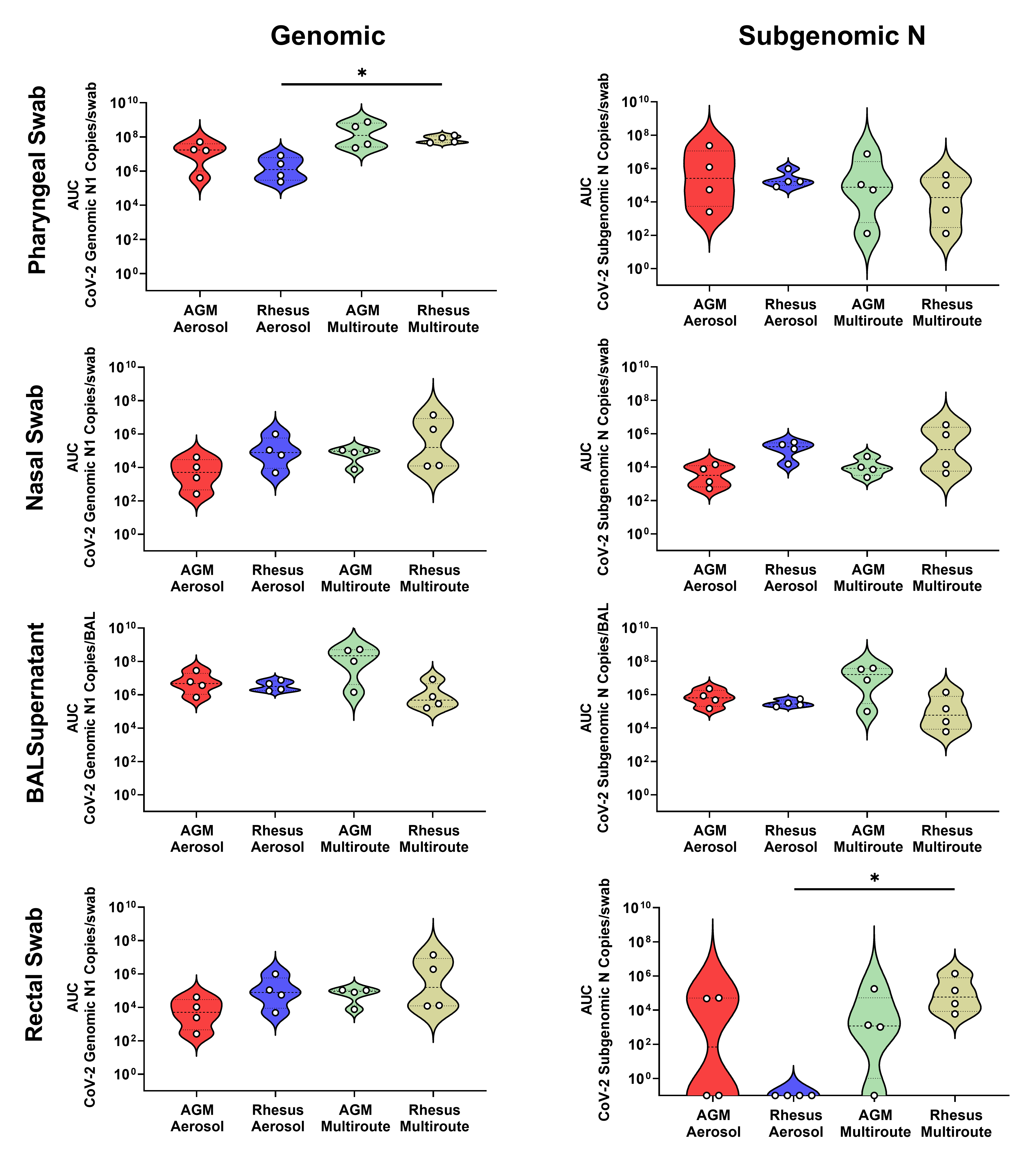

Supplement: S1 Fig — Viral loads, assessed by RT-qPCR for genomic and subgenomic RNA, represented as area under the curve for the post challenge period. Comparisons between groups were made via Kruskal-Wallis with Dunn’s multiple comparisons test. Asterisks represent significant comparisons (*, p<0.05). (TIF) [file ppat.1010618.s001.tif]

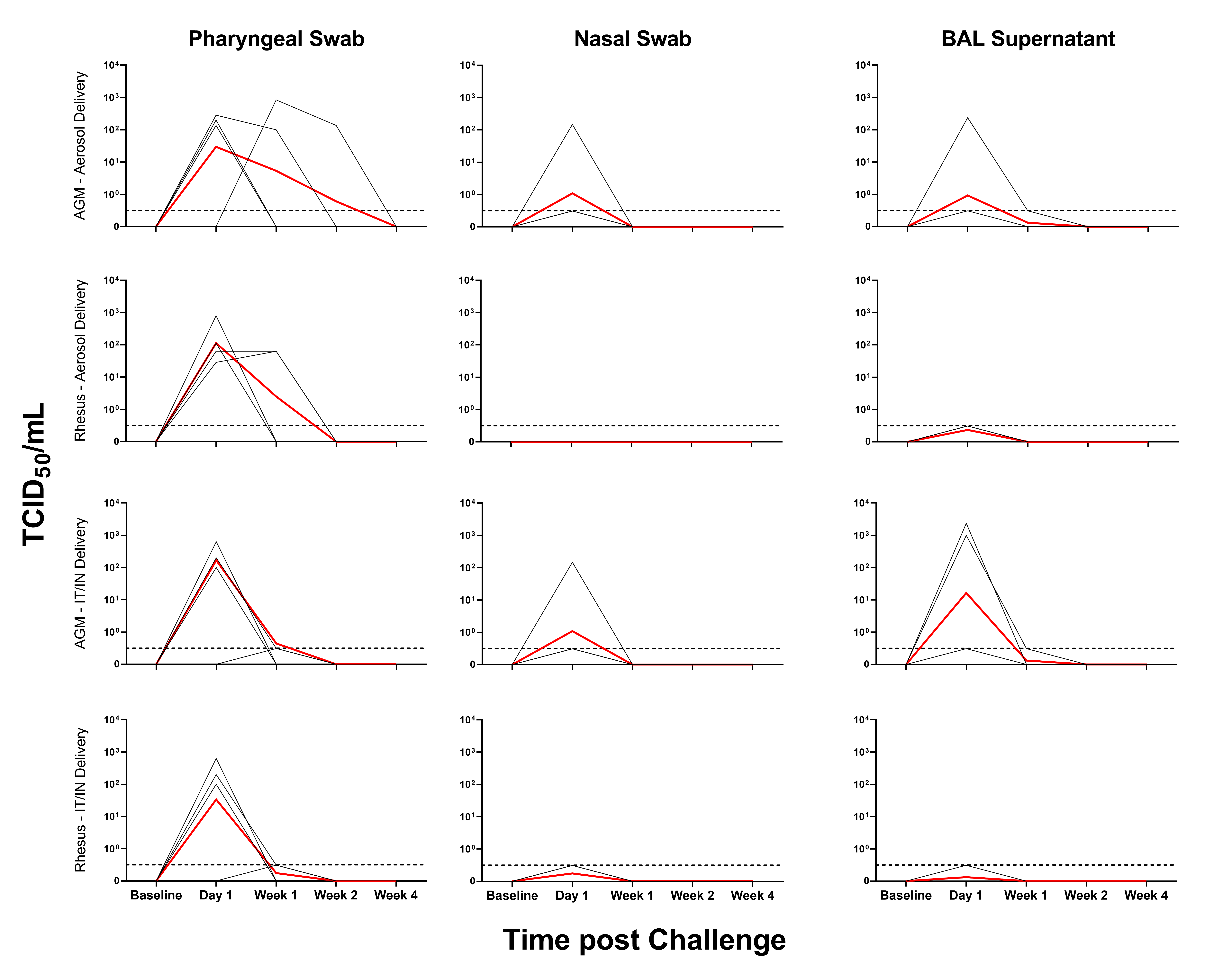

Supplement: S2 Fig — Viral loads, assessed by TCID50, represented as area under the curve for the post challenge period. Black lines indicate viral loads per individual, with red lines indicating group geometric means. Dotted lines indicate a positive sample below the limit of quantification. (TIF) [file ppat.1010618.s002.tif]

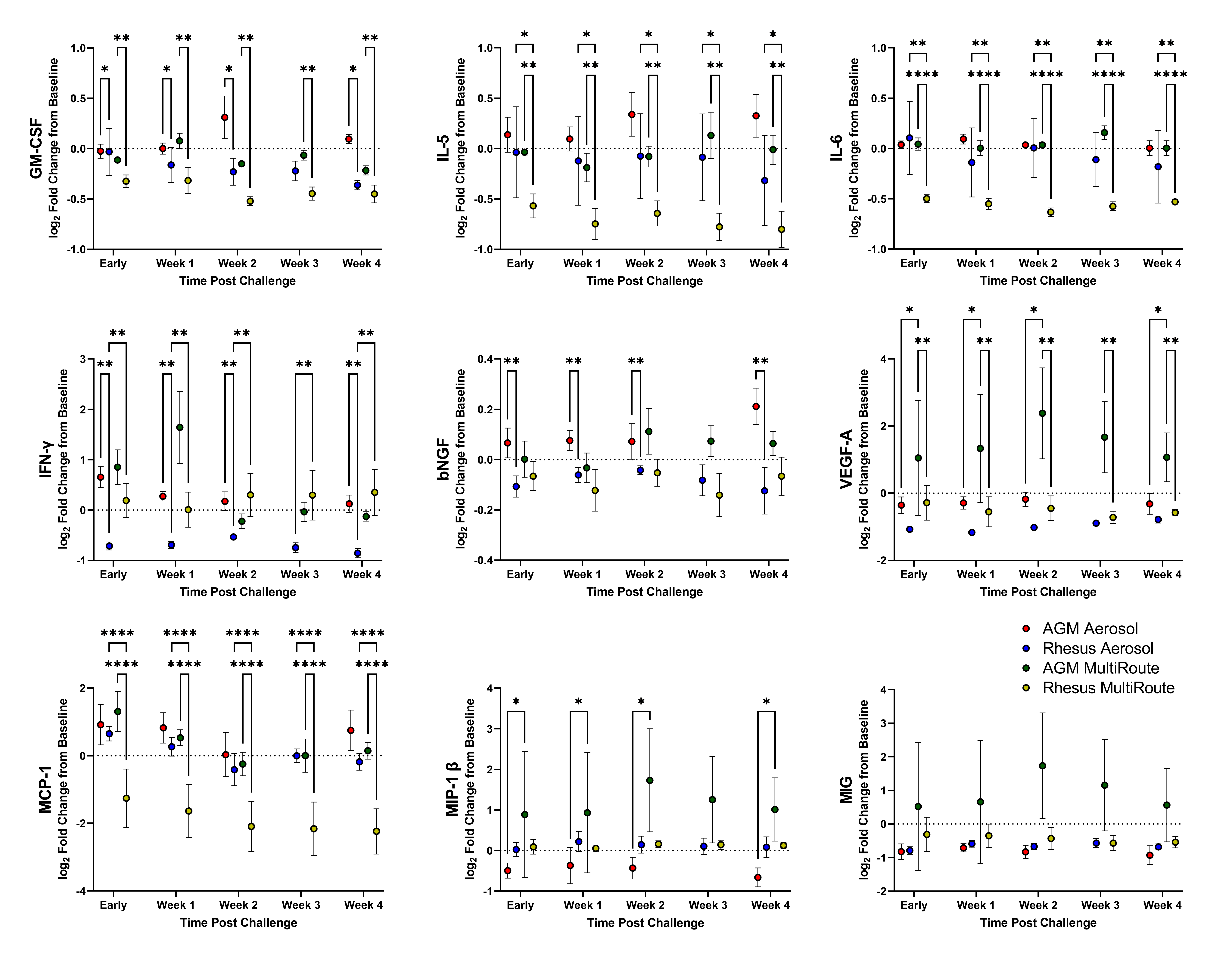

Supplement: S3 Fig — Cytokines circulating in serum were analyzed at indicated time points post challenge, with early indicating a mean value of days 1, 2 and 3 post challenge. Comparisons were made with two-way ANOVA using Tukey’s multiple comparisons test. Asterisks represent significant comparisons (*, p<0.05; **, p<0.01;****, p<0.0001). (TIF) [file ppat.1010618.s003.tif]

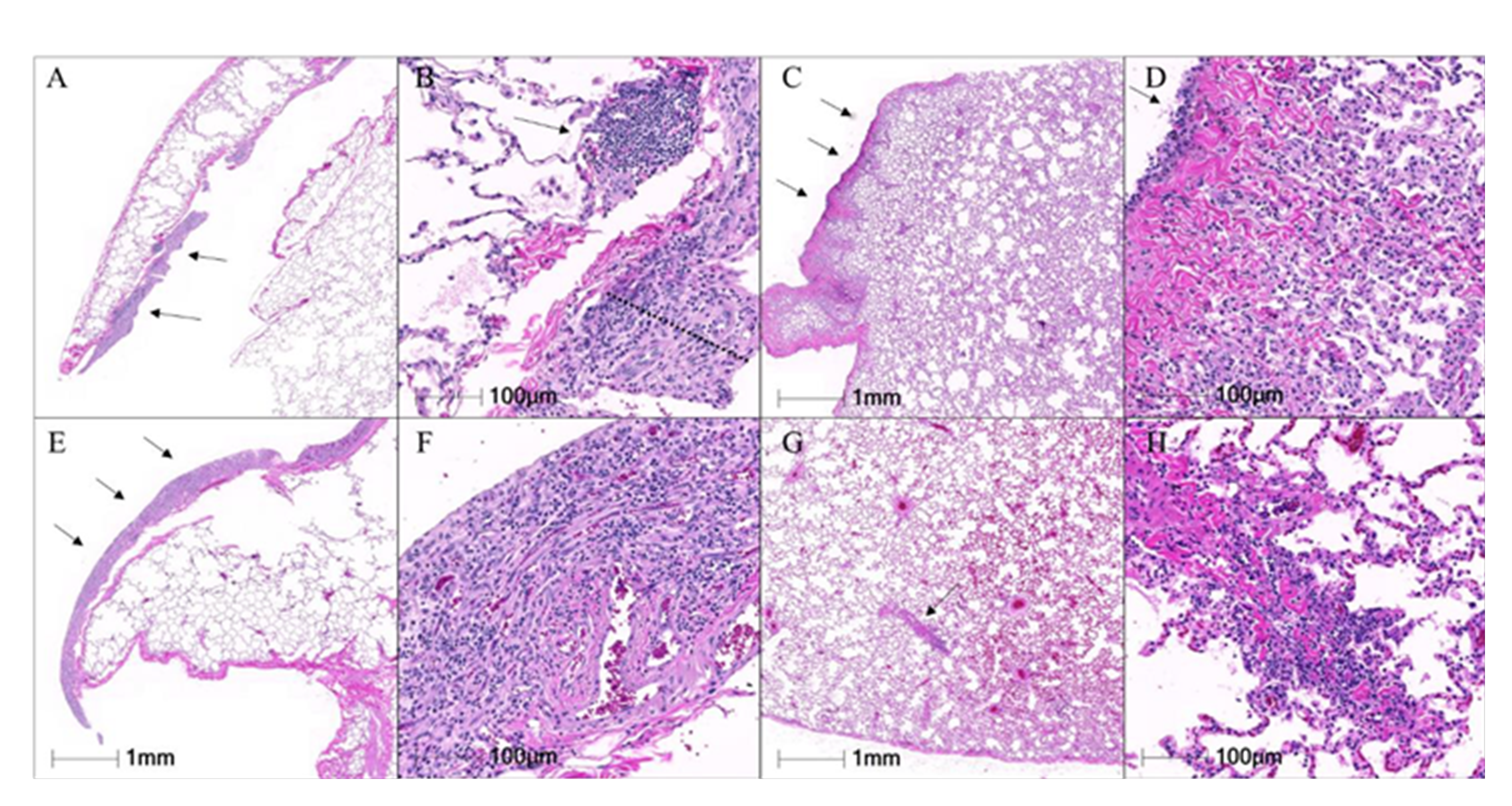

Supplement: S4 Fig — A,B: Aerosol RM, right middle lobe. A) The pleura is segmentally thickened (pleuritis, arrows). B) Regions of pleuritis are characterized by fibrosis (dotted line) with infiltration by mononuclear cells. Aggregates of similar inflammatory cells are present subpleurally (arrow). C,D: Aerosol AGM, left anterior lobe. C) The pleura is segmentally thickened (arrows). D) The pleura is lined by hypertrophic mesothelial cells (arrow) and there is infiltration of the subpleural parenchyma by histiocytes. E,F: IT/IN RM, left lower lobe. E) The pleura is segmentally thickened (pleuritis, arrows). F) The pleura is thickened by fibrosis and infiltrated by mononuclear cells, predominately lymphocytes. G,H: IT/IN AGM, right lower lobe. G) There is mild congestion and rare perivascular inflammation (arrow). H) Perivascular inflammation is characterized by infiltration of the tunica adventitia by mononuclear cells. (TIF) [file ppat.1010618.s004.tif]

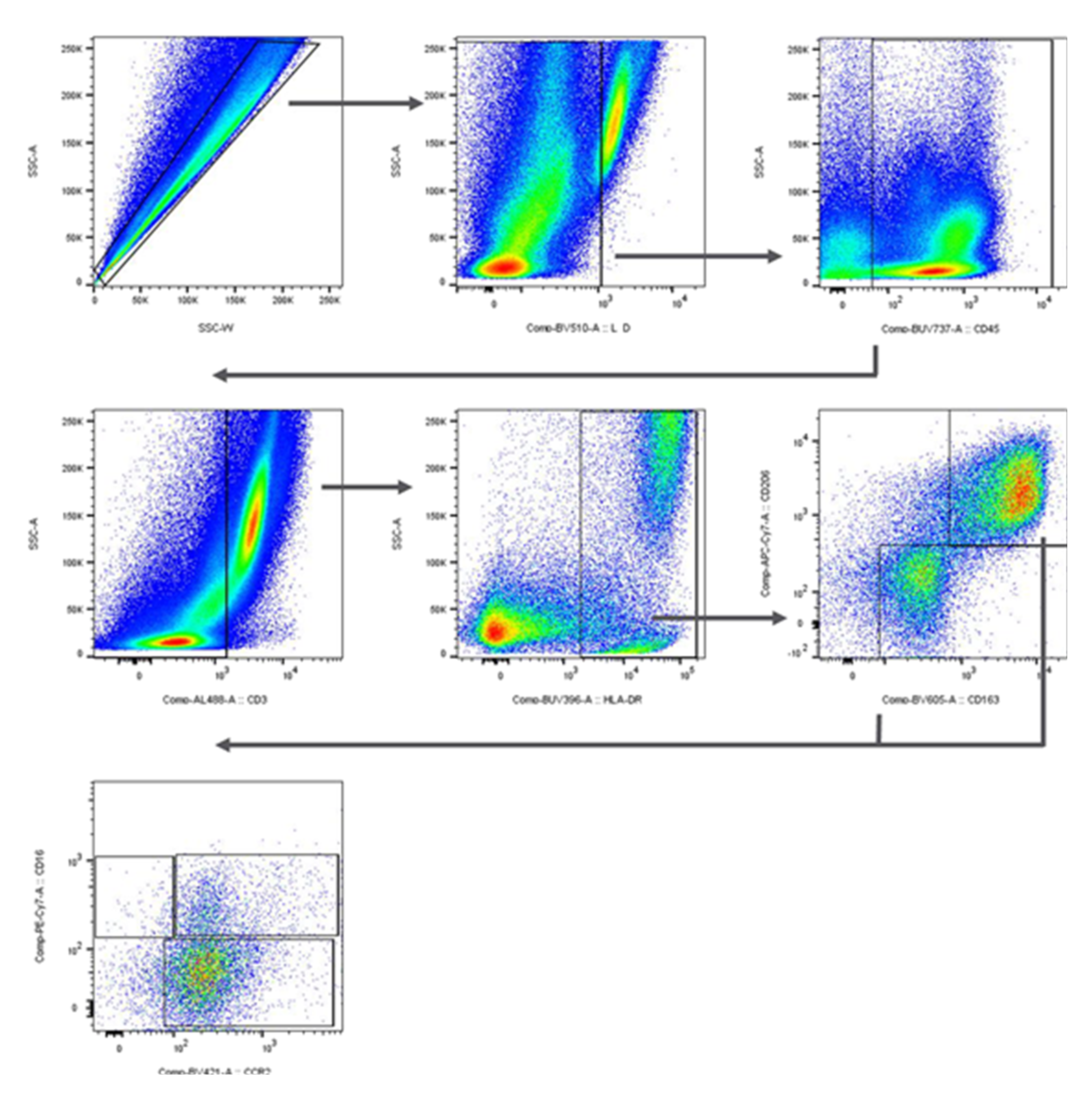

Supplement: S5 Fig — Representative gating strategy to classify alveolar (CD163+CD206+), interstitial (CD163+CD206-), monocyte-derived (CD163+CD206+CD16+CCR2+), and resident alveolar (CD163+CD206+CD16-) macrophages in BAL. (TIF) [file ppat.1010618.s005.tif]

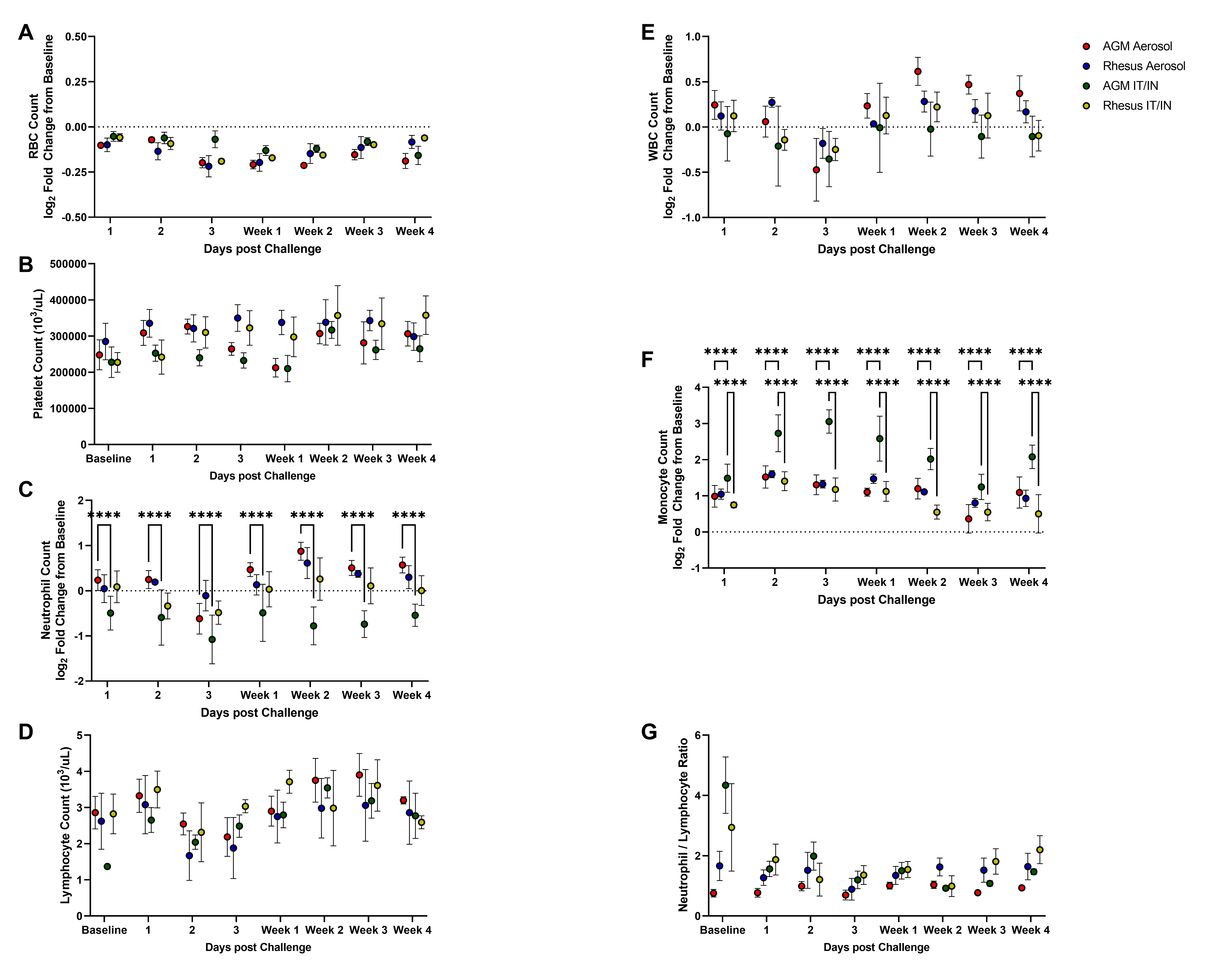

Supplement: S6 Fig — Complete blood counts were performed at indicated times and were compared for counts of RBCs, platelets, neutrophils, lymphocytes, WBCs and monocytes (A, B, C, D, E, and F, respectively), as well as neutrophil/lymphocytes ratio (G). Comparisons were made via two-way ANOVA with Tukey’s multiple comparisons test. Asterisks represent significant comparisons (****, p<0.0001). (TIF) [file ppat.1010618.s006.tif]

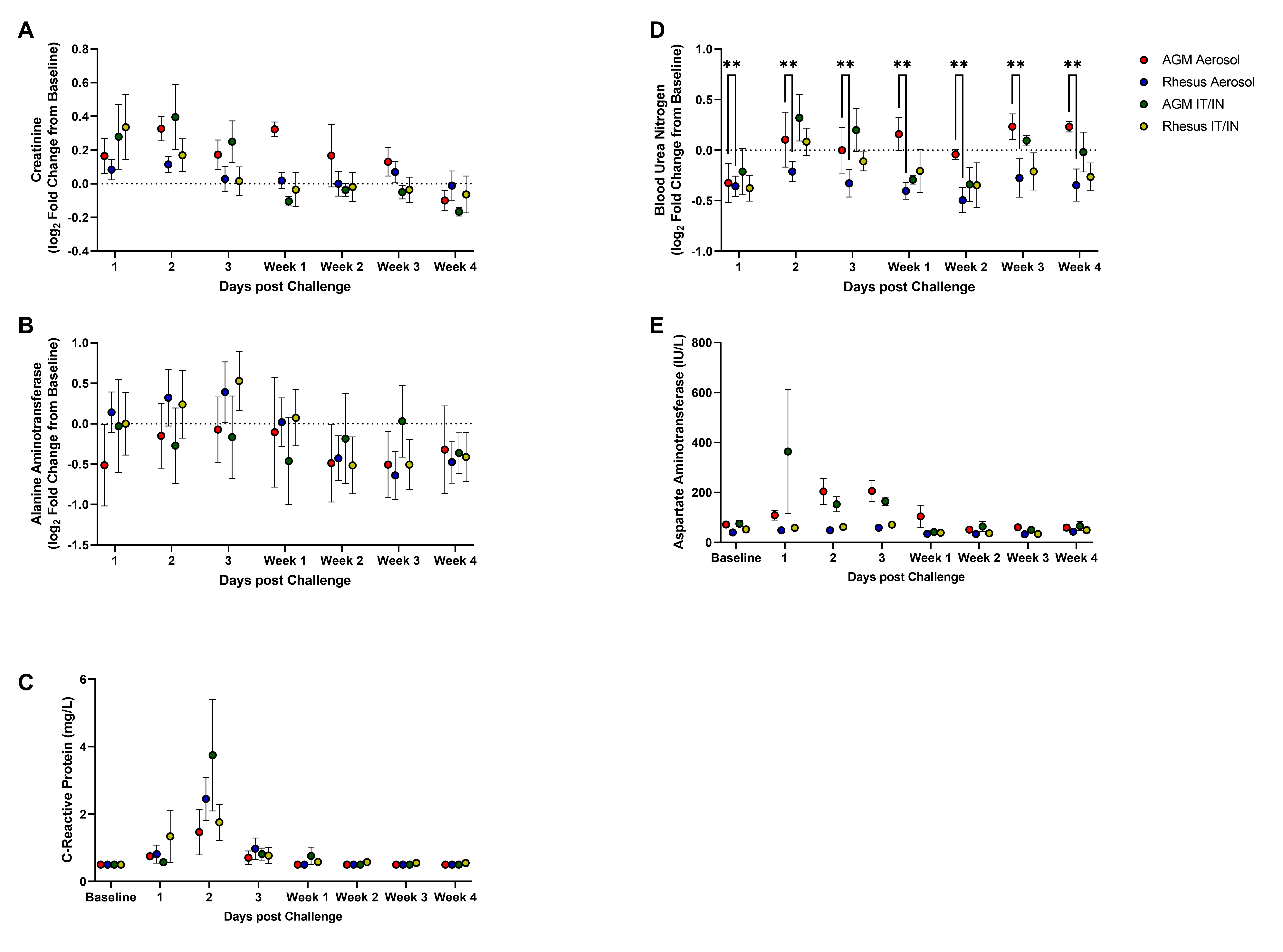

Supplement: S7 Fig — Clinical chemistries were performed at the indicated times post challenge. Comparisons between each group were made for log2 fold change from baseline of creatinine (A), ALT (B), BUN (D) and concentrations of CRP (C) and AST (E). Comparisons were made via two-way ANOVA with Tukey’s multiple comparisons test. Asterisks represent significant comparisons (**, p<0.01). (TIF) [file ppat.1010618.s007.tif]

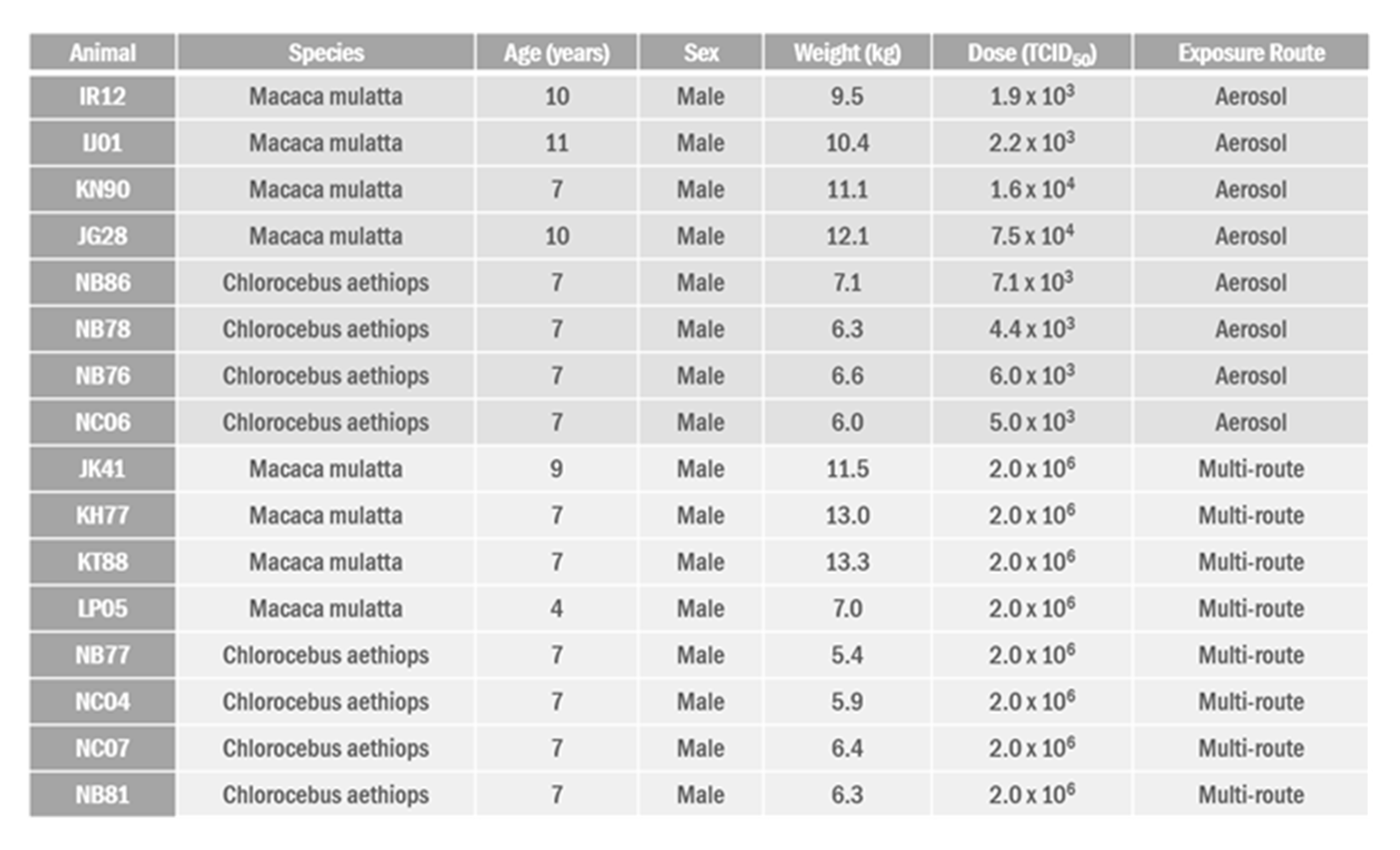

Supplement: S1 Table — (TIF) [file ppat.1010618.s008.tif]

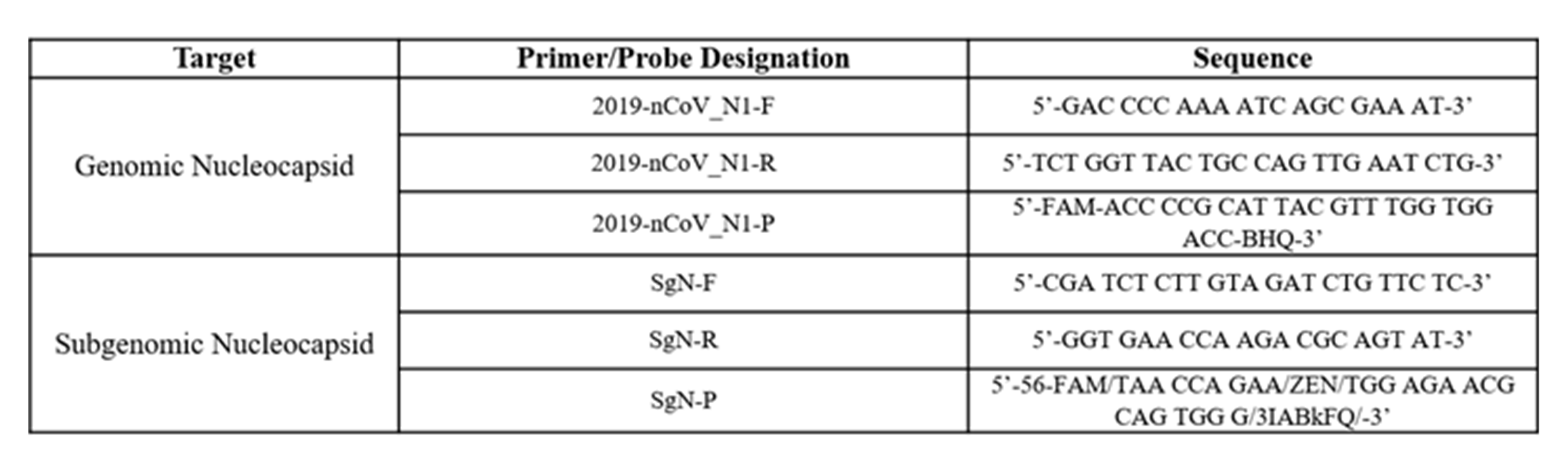

Supplement: S2 Table — (TIF) [file ppat.1010618.s009.tif]

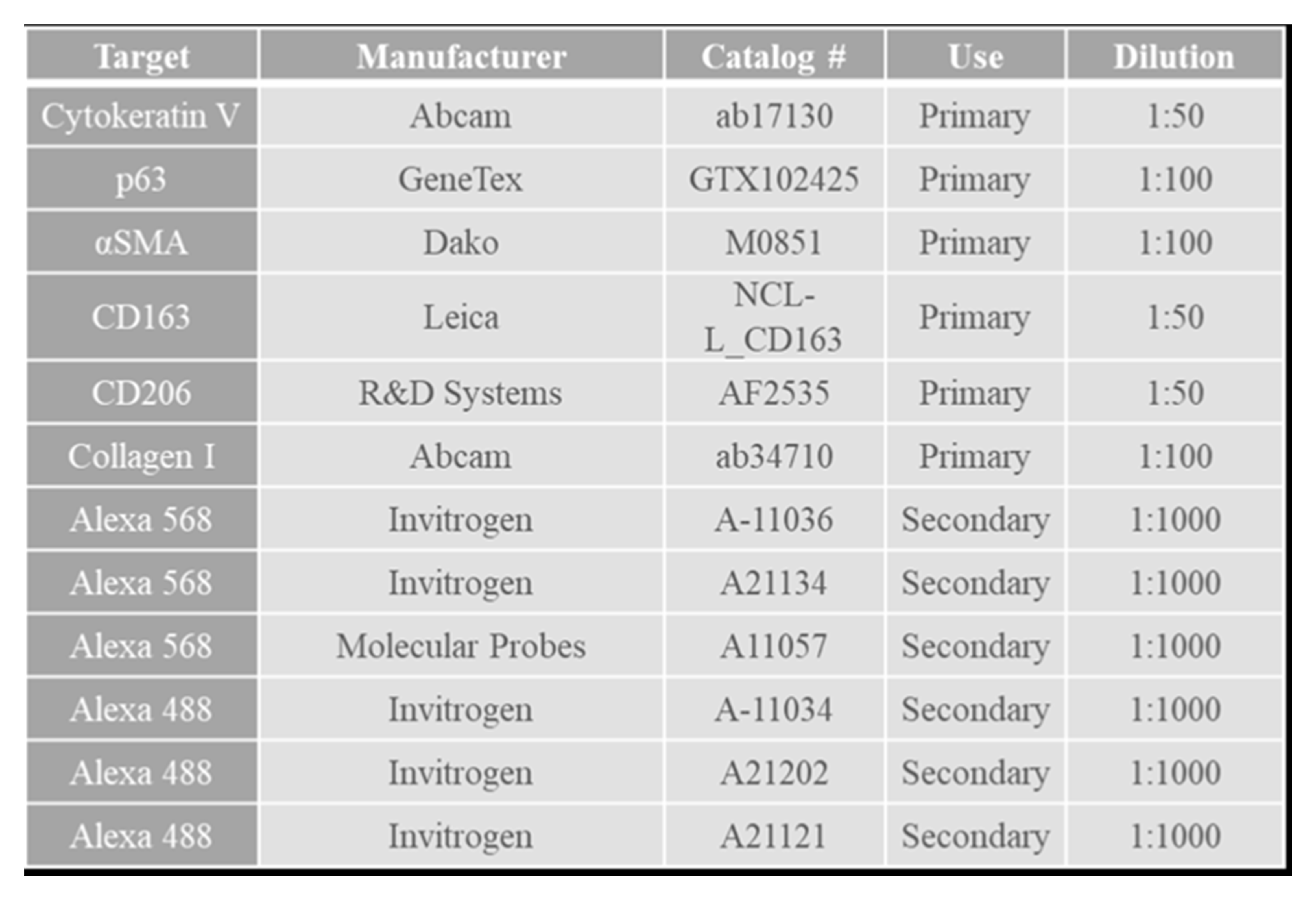

Supplement: S3 Table — (TIF) [file ppat.1010618.s010.tif]

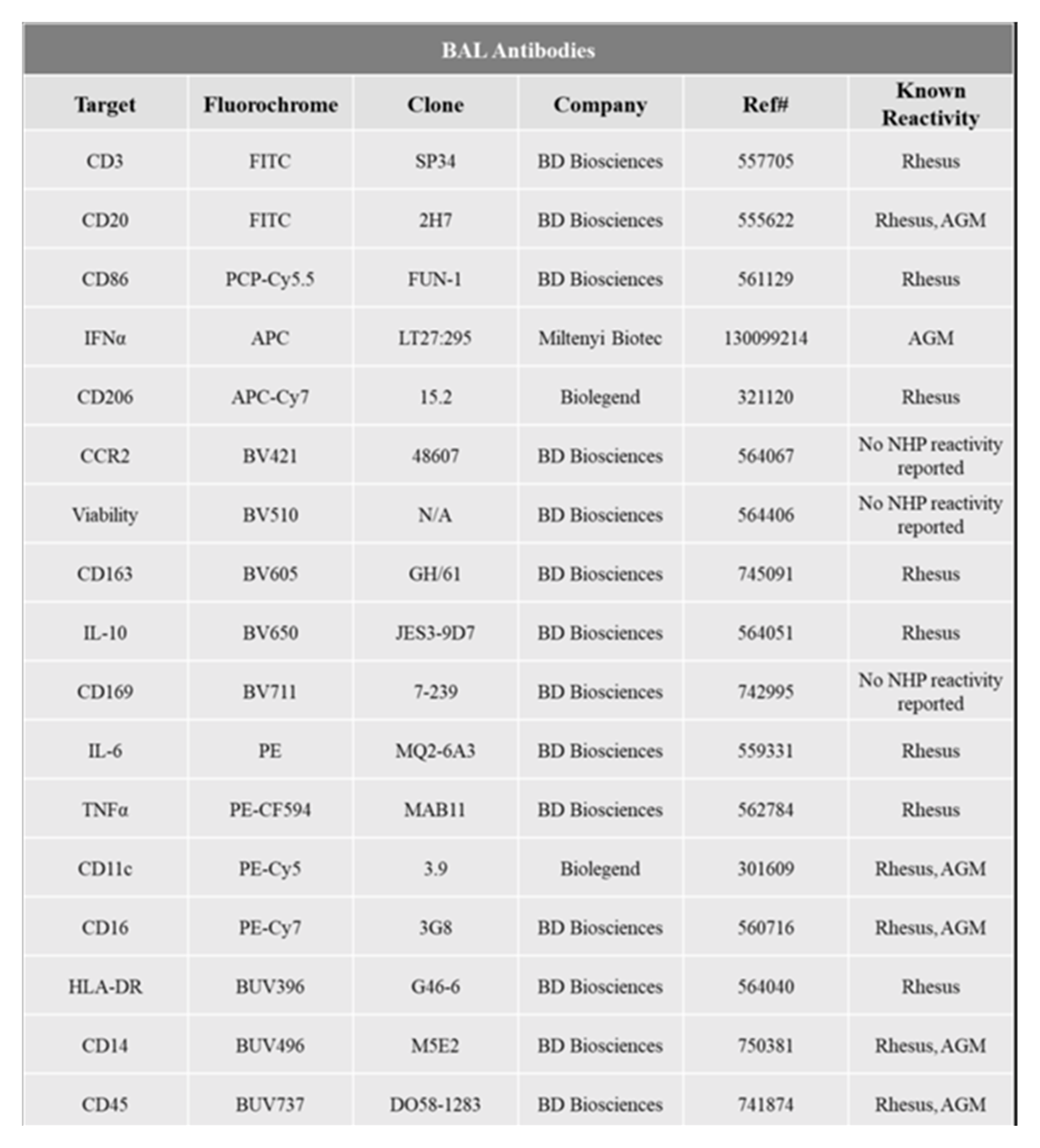

Supplement: S4 Table — (TIF) [file ppat.1010618.s011.tif]
